# Assessments of dietary intake and polygenic risk score in associations with colorectal cancer risk: evidence from the UK Biobank

**DOI:** 10.1186/s12885-023-11482-1

**Published:** 2023-10-18

**Authors:** Tung Hoang, Sooyoung Cho, Ji-Yeob Choi, Daehee Kang, Aesun Shin

**Affiliations:** 1 Department of Preventive Medicine, Seoul National University College of Medicine, Seoul, Korea; 2 Integrated Major in Innovative Medical Science, Seoul National University Graduate School, Seoul, Korea; 3 Genomic Medicine Institute, Medical Research Center, Seoul National University, Seoul, Korea; 4 Department of Biomedical Sciences, Seoul National University Graduate School, Seoul, Korea; 5 BK21plus Biomedical Science Project, Seoul National University College of Medicine, Seoul, Korea; 6 Institute of Health Policy and Management, Medical Research Center, Seoul National University, Seoul, Korea; 7 Cancer Research Institute, Seoul National University, Seoul, Korea; 8 Institute of Environmental Medicine, Medical Research Center, Seoul National University, Seoul, Korea

**Keywords:** Diet, Polygenic risk score, Colorectal cancer, Interaction

## Abstract

**Background:**

This study aimed to explore the potential interaction between dietary intake and genetics on incident colorectal cancer (CRC) and whether adherence to healthy dietary habits could attenuate CRC risk in individuals at high genetic risk.

**Methods:**

We analyzed prospective cohort data of 374,004 participants who were free of any cancers at enrollment in UK Biobank. Dietary scores were created based on three dietary recommendations of the World Cancer Research Fund (WCRF) and the overall effects of 11 foods on CRC risks using the inverse-variance (IV) method. Genetic risk was assessed using a polygenic risk score (PRS) capturing overall CRC risk. Cox proportional hazard models were used to calculate hazard ratios (HRs) and 95% CIs (confidence intervals) of associations. Interactions between dietary factors and the PRS were examined using a likelihood ratio test to compare models with and without the interaction term.

**Results:**

During a median follow-up of 12.4 years, 4,686 CRC cases were newly diagnosed. Both low adherence to the WCRF recommendations (HR = 1.12, 95% CI = 1.05–1.19) and high IV-weighted dietary scores (HR = 1.27, 95% CI = 1.18–1.37) were associated with CRC risks. The PRS of 98 genetic variants was associated with an increased CRC risk (HR_T3vsT1_ = 2.12, 95% CI = 1.97–2.29). Participants with both unfavorable dietary habits and a high PRS had a more than twofold increased risk of developing CRC; however, the interaction was not significant. Adherence to an overall healthy diet might attenuate CRC risks in those with high genetic risks (HR = 1.21, 95% CI = 1.08–1.35 for high vs. low IV-weighted dietary scores), while adherence to WCRF dietary recommendations showed marginal effects only (HR = 1.09, 95% CI = 1.00–1.19 for low vs. high WCRF dietary scores).

**Conclusion:**

Dietary habits and the PRS were independently associated with CRC risks. Adherence to healthy dietary habits may exert beneficial effects on CRC risk reduction in individuals at high genetic risk.

**Supplementary Information:**

The online version contains supplementary material available at 10.1186/s12885-023-11482-1.

## Introduction

According to reports from the Global Cancer Observatory 2020, colorectal cancer (CRC) is the third most common cancer worldwide [1]. It is estimated that there will be approximately 1.9 million new CRC cases in 2020, and that number is predicted to increase to 3.2 million CRC new cases in 2040 [1]. Colorectal carcinogenesis is strongly promoted by oxidative stress and chronic inflammation via reactive oxygen species and proinflammatory cytokines [2]. In addition, diets rich in antioxidants and anti-inflammatory factors have shown inverse associations with CRC development [3, 4]. Furthermore, diets may indirectly affect colorectal carcinogenesis risk via CRC risk factors (e.g., obesity) and the gut microbiota [5].

Given the contribution of genetic factors to the development of CRC [6], previous studies investigated the effect of dietary intake on CRC risks according to susceptibility loci [7, 8]. In the Genetics of Colorectal Cancer Consortium and Colon Cancer Family Registry, the interaction between 10 CRC susceptibility SNPs was evaluated with several dietary factors, including red meat, processed meat, fruit, vegetables, and alcohol consumption [7]. Among 10 genetic variants, only rs16892766 was found to interact with vegetable intake [7]. However, one single nucleotide polymorphism (SNP) may be limited in reflecting the overall genetic risk of CRC. With widespread genome-wide association studies (GWASs), subsequent research has examined whether the association between diet consumption and CRC risk differed according to CRC susceptibility status [9]. Genetic variants identified from GWAS vary from low penetrance (common variants) to moderate and high penetrance (rare variants) [10, 11]. Thus, combining multiple SNPs into a single polygenic risk score (PRS) is an alternative approach to reflect the overall genetic predisposition to CRC [12, 13].

In the concept of nutrigenetics, genetic factors may impact the effect of diets on health outcomes by altering the bioactivity of metabolic pathways and mediators [14]. To date, the extent to which the CRC risk of individuals with a high overall genetic risk can be improved by adherence to a healthy dietary habit remains unclear. Previous studies have reported interindividual variability in responses to the same dietary factors [15]. Identical meals were shown to largely contribute to postprandial responses of triglycerides, whereas overall genetic factors did not explain any variances in triglyceride responses [16]. However, a recent study revealed a relationship between genetically predicted polyunsaturated fatty acids (PUFAs) and CRC risk [17]. Thus, we hypothesized that the effect of dietary intake on CRC risks may differ by genetic factors associated with CRC.

Furthermore, recent dietary guidelines have shifted the focus from single food items to the overall diet, which considers the complex interrelationships among different foods and reflects individuals’ actual dietary habits [18]. Therefore, while hypothesizing that pleiotropic pathways, rather than the individual effects of each exposure, may be the underlying cause of the increased or reduced effect of dietary and genetic factors, we conducted this study to explore the associations of dietary intake with CRC risk and explore the joint effect of dietary factors and genetic factors contributing to the incidence of CRC by constructing a PRS using data from the largest-to-date GWAS [10, 11]. Additionally, by hypothesizing that genetic predisposition may alter the association of diets with CRC risk, we identified dietary factors that may attenuate CRC incidence in individuals at high genetic risk.

## Materials and methods

### Study design and data collection

We carried out a prospective cohort study of participants recruited from UK Biobank. A detailed description of the study design is available elsewhere [19–21]. Overall, eligible participants were recruited from 22 assessment centers across England, Wales, and Scotland between 2006 and 2010. All the study participants provided electronically signed consent using a signature-capture device. Information on demographics, lifestyles, and medication history was collected via a touchscreen questionnaire. This touchscreen questionnaire was also used to inquire about habitual diet consumption in the preceding year [22]. Anthropometric factors were measured following standardized procedures.

**Fig. 1:**
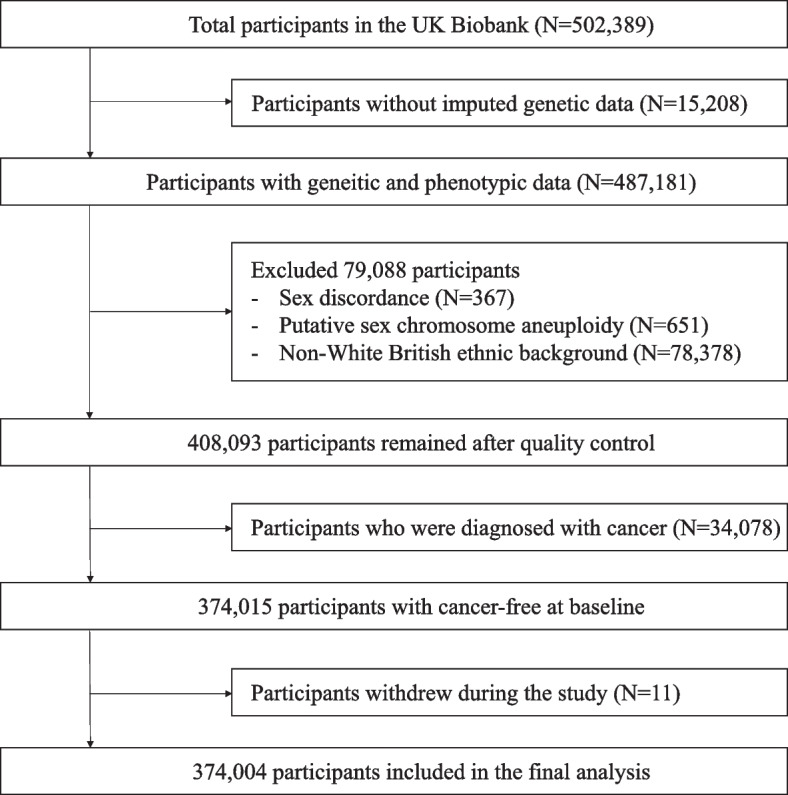
Flowchart for the study participants

Of the 502,389 participants recruited, we excluded individuals with no genetic information (*N* = 15,208). We further excluded those with sex discordance (*N* = 367), putative sex chromosome aneuploidy (*N* = 651), and ethnic backgrounds other than White British (*N* = 78,378). Of 408,093 participants remaining after quality control, 374,004 participants who were free of any cancers at baseline and did not withdraw during the study were eligible for the final analysis (Fig. 1).

### Dietary information

In this study, habitual food intake was assessed via a touchscreen questionnaire [22]. Participants were asked about the frequency of consumption of oily and nonoily fish, processed meat, beef, lamb/mutton, pork, poultry, and cheese. The frequency was specified as follows: less than once a week, once a week, 2–4 times a week, 5–6 times a week, and once or more daily. Participants were also asked about the intake of cooked and salad/raw vegetables (tablespoons/day), fresh and dried fruit (pieces/day), and coffee and tea (cups/day). For alcohol consumption, participants were asked to choose one of the following intake frequencies: daily or almost daily, three or four times a week, once or twice a week, one to three times a month, special occasions only, and never. Any answers with ‘do not know’ or ‘prefer not to answer’ were considered missing. We grouped single items to obtain the consumption of red meat (times/week), total fish (times/week), total fruit (servings/day), and total vegetables (servings/day) [23]. Daily milk consumption (mL/day) was estimated based on information on the type of milk, and the numbers of bowls of breakfast cereal, cups of coffee, and cups of tea consumed [23]. The cutoffs for categories of food groups and food items were chosen based on the distribution of food frequencies. A summary of the touchscreen questionnaire for food items included in the analysis is available in Additional file 1: Table S1.

To capture the overall dietary habits, we calculated a World Cancer Research Fund (WCRF) dietary score based on the extent to which participants adhered to the dietary recommendations. This score was determined by counting the number of dietary components followed by each participant. The simplified-WCRF/American Institute for Cancer Research (AICR) 2018 score and the American Cancer Society Guidelines on Nutrition and Physical Activity for Cancer Prevention were obtained. Accordingly, the WCRF dietary score was calculated based on the adherence to three dietary recommendations, including consumption of red and processed meat less than 4 times/week, fruit and vegetable intake greater than 5 servings/day, and alcohol consumption less than 3 times/month [24, 25]. Each dietary recommendation was treated as a binary variable with a value of 1 assigned to adherence and a value of 0 assigned to nonadherence. We also created an IV-weighted dietary score by adding the natural logarithm of hazard ratio (HR) divided by the corresponding standard error for the estimate of each dietary factor in association with CRC. This approach may reflect both the strength and variation of the effect of dietary factors. Any dietary factors (red meat, processed meat, poultry, fish, milk, cheese, fruit, vegetables, coffee, tea, and alcohol), which were reported by the WCRF/AICR, regardless of the level of evidence, were included in the calculation [26]. The distribution of participants at different levels of adherence to dietary recommendations was as follows: score = 0 (*N* = 42,599), score = 1 (*N* = 127,264), score = 2 (153,729), and score = 3 (*N* = 47,310). Thus, we divided participants into two groups by the WCRF dietary score (0–1 and 2–3) and three groups by the IV-weighted dietary score (tertiles: -13.22 to < -2.88, -2.88 to < 1.09, and 1.09 to 13.02) to have similar numbers of participants in each group. Accordingly, higher WCRF and lower IV-weighted dietary scores indicated healthier diets, and vice versa.

### Genotyping and polygenic risk score

Individuals were genotyped using the custom UK Biobank Axiom Array and the Affymetrix Axiom Array, which capture 805,426 markers, as described elsewhere [20]. Both the UK10K and 1000 Genomes Phase 3 and the Haplotype Reference Consortium reference panel were used to imputed genotyping data, which resulted in a total of 93,095,623 markers [20].

Susceptibility loci for CRC risk were derived from two large meta-analyses of GWAS in European populations [10, 11]. Utilizing whole-genome sequencing data for 1,439 CRC and 720 controls from five studies and GWAS data for 58,131 CRC or advanced adenoma cases and 67,347 controls from 45 studies in GECCO, CORECT, and CCFR, Huyghe discovered 40 novel independent SNPs [10]. Using GWAS data for 31,197 cases and 61,770 controls from 15 studies from CCFR1, CCFR2, COIN, CORSA, Croatia, DACHS, FIN, NSCCG-OncoArray, SCOT, Scotland1, SOCCS/GS, SOCCS/LBC, UK1, and UK Biobank, Law identified 31 additional independent SNPs [11]. Taken together with known genetic variants, a total of 221 SNPs were identified [27]. After excluding duplicate, missing, ambiguous, and high linkage disequilibrium variants, 127 SNPs remained [27]. By excluding UK Biobank data, beta-coefficients and standard errors for the effect estimate of these variants on CRC were recalculated to avoid bias and overlap [27]. Of the 127 variants for CRC susceptibility, 78 SNPs were available in the imputed UK Biobank data. We identified the closest SNPs determined to be in linkage disequilibrium (LD) with 49 unavailable SNPs. Of these, 20 SNPs with r2 greater than 0.8 for LD coefficients were considered good proxies and included in the calculation of the PRS. The proxy SNP selection was performed using the LDproxy tool, with the reference data panel of European ancestry [28, 29]. Thus, a total of 98 SNPs were used to calculate the PRS (Additional file 1:Table S2).

The PRS calculation was considered according to three approaches [30]. First, we used an unweighted PRS $({PRS}_{unw})$, which corresponds to the sum of the number of effect alleles. Second, we used a standard weighted PRS $({PRS}_{\beta })$, which added the log odds ratio (β) for each effect allele as weights. Third, we used an inverse variance (IV)-weighted PRS $({PRS}_{IV})$, in which the weights incorporated both the log odds ratio (β) and standard error (SE) of effect alleles.

$${PRS}_{unw}={\sum }_{i=1}^{98}{SNP}_{i};{PRS}_{\beta }={\sum }_{i=1}^{98}{\beta }_{i}*{SNP}_{i};{PRS}_{IV}={\sum }_{i=1}^{98}\frac{{\beta }_{i}}{{SE}_{i}}*{SNP}_{i}$$

### Outcome ascertainment

The primary outcome was CRC incidence, in which the diagnoses were defined by the International Classification of Disease 10 codes. CRC was defined as either colon cancer (C18.0-C18.9) or rectal cancer (C19 and C20). The follow-up time was defined from the date of study participation to the date of CRC diagnosis, death, loss-to-follow-up, or end of follow-up (June 25, 2021), whichever came first.

### Statistical analysis

The characteristics and diet consumption are presented as the mean ± standard deviation for continuous variables and counts (percentage) for categorical variables. Univariate analysis was performed for demographic and lifestyle factors using the Cox regression model, and factors associated with CRC risk were identified (Additional file 1: Table S3). Accordingly, sex, first-degree family history of CRC, household income, smoking status, alcohol consumption, body mass index (BMI), and physical activity were adjusted in the multivariable analysis of dietary intake and CRC risk.

To estimate the effect of the PRS, the association between each tertile and decile of the PRS with CRC was estimated using the Cox regression model adjusting for sex and family history of CRC. The interactions between food consumption and the IV-weighted PRS, which upweighted the contribution of variants with more precisely estimated effects, were assessed. We modeled the dietary factor and the PRS independently and tested their interactions by comparing the model with a model additionally adjusted for the PRS*diet interaction term, using the likelihood ratio test. Since UK Biobank participants were aged 39 to 73 years, we calculated the adjusted cumulative risk of developing CRC at the age of 80 years for individuals of each combination of dietary and PRS categories. The cumulative risk was defined as the complement of the cumulative survival adjusted for covariates. We further examined the association between dietary intake and CRC risk stratified by PRS tertiles to explore the benefit of adherence to a healthy diet with CRC risk among individuals of different genetic risk profiles.

To quantify the contribution of dietary intake or genetic risk to CRC incidence, we calculated the attributable fraction, which is the estimated proportional reduction in CRC incidence that would occur if all had been unexposed to the risk factor for interest [31]. We assumed that the prevalence of exposure in our study would reflect the prevalence of exposure in the general European population.

Subgroup analyses were conducted according to sex (men and women) and anatomical cancer subsites (colon and rectal cancer). Missing data were handled in a complete-case analysis approach, where the analysis restricted to participants with complete information on all variables included in the model [32]. Quality controls for genotyping data were performed in PLINK [33], and statistical analyses were implemented using R version 4.1.2 (Foundation for Statistical Computing, Vienna, Austria).

## Results

### Demographic and lifestyle characteristics of study participants

After a median follow-up of 12.4 years (interquartile range 11.6–13.0), 4,686 participants were diagnosed with CRC (3,131 colon cancer and 1,555 rectal cancer incident cases). Participants had a mean (standard deviation) age of 56.6 (8.0) years, and 199,428 (53.3%) were women. Information on other characteristics, including family history of CRC, household income, smoking status, alcohol consumption, BMI, and physical activity, is also shown in Table 1.

**Table 1:** General characteristics of study participants

Factor	Total (*N* = 374,004)	Low PRS	Intermediate PRS	High PRS
**Age (years)**	56.6 ± 8.0	56.7 ± 8.0	56.7 ± 8.0	56.6 ± 8.0
39 to < 50 years	84,953 (22.7)	28,305 (22.7)	28,323 (22.7)	28,325 (22.7)
50 to < 60 years	125,014 (33.4)	41,547 (33.3)	41,581 (33.4)	41,886 (33.6)
60 to ≤ 73 years	164,037 (43.9)	54,816 (44.0)	54,764 (43.9)	54,457 (43.7)
**Sex**				
Women	199,425 (53.3)	66,126 (53.0)	66,596 (53.4)	66,703 (53.5)
Men	174,579 (46.7)	58,542 (47.0)	58,072 (46.6)	57,965 (46.5)
**First-degree family history of CRC**				
No	271,917 (72.7)	91,490 (73.4)	90,807 (72.8)	89,620 (71.9)
Yes	26,499 (7.1)	7,709 (6.2)	8,710 (7.0)	10,080 (8.1)
Missing	75,588 (20.2)	25,469 (20.4)	25,151 (20.2)	24,968 (20.0)
**Household income (£/year)**				
≤ 31,000	152,984 (40.9)	50,835 (40.8)	50,869 (40.8)	51,280 (41.1)
31,000 to < 52,000	85,769 (22.9)	28,592 (22.9)	28,619 (23.0)	28,558 (22.9)
≥ 52,000	83,916 (22.4)	28,208 (22.6)	28,094 (22.5)	27,614 (22.2)
Missing	51,335 (13.7)	17,033 (13.7)	17,086 (13.7)	17,216 (13.8)
**Smoking status**				
Never	204,510 (54.7)	68,611 (55.0)	67,826 (54.4)	68,073 (54.6)
Former	130,240 (34.8)	43,104 (34.6)	43,721 (35.1)	43,415 (34.8)
Current	37,976 (10.2)	12,536 (10.1)	12,728 (10.2)	12,712 (10.2)
Missing	1,278 (0.3)	417 (0.3)	393 (0.3)	468 (0.4)
**Alcohol consumption**				
Never or rarely	63,285 (16.9)	21,083 (16.9)	21,160 (17.0)	21,042 (16.9)
Once a month to twice a week	140,555 (37.6)	46,746 (37.5)	46,886 (37.6)	46,923 (37.6)
3–4 times/week	90,746 (24.3)	30,434 (24.4)	29,993 (24.1)	30,319 (24.3)
Daily or more	79,157 (21.2)	26,320 (21.1)	26,533 (21.3)	26,304 (21.1)
Missing	261 (0.1)	85 (0.1)	96 (0.1)	80 (0.1)
**BMI (kg/m**^**2**^**)**	27.4 ± 4.8	27.4 ± 4.7	27.4 ± 4.8	27.4 ± 4.8
< 25.0 kg/m^2^	12,2550 (32.8)	40,787 (32.7)	40,843 (32.8)	40,920 (32.8)
25.0 to < 30 kg/m^2^	159,756 (42.7)	53,447 (42.9)	53,203 (42.7)	53,106 (42.6)
≥ 30 kg/m^2^	90,528 (24.2)	30,046 (24.1)	30,222 (24.2)	30,260 (24.3)
Missing	1,170 (0.3)	388 (0.3)	400 (0.3)	382 (0.3)
**Moderate or vigorous activity**				
Insufficient	137,879 (36.9)	45,770 (36.7)	46,056 (36.9)	46,053 (36.9)
Sufficient	165,319 (44.2)	55,339 (44.4)	54,983 (44.1)	54,997 (44.1)
Missing	70,806 (18.9)	23,559 (18.9)	23,629 (19.0)	23,618 (18.9)

*PRS* polygenic risk score, *BMI* body mass index. Low PRS: 316 to < 454; intermediate PRS: 454 to < 483; high PRS: 483 to ≤ 621. Data are presented as mean ± standard deviation for continuous variables and count (percentage) for categorical variables

### Dietary intakes and associations with colorectal cancer

Table 2 presents the habitual intake in association with CRC risk. Having a low adherence to the WCRF (HR = 1.12, 95% CI = 1.05–1.09) and a high IV-weighted dietary score (HR = 1.27, 95% CI = 1.18–1.37) was significantly associated with increased risks of CRC. Regarding dietary components, those who consumed ≥ 3 times per week had a 16% higher risk of CRC (HR = 1.16, 95% CI = 1.08–1.25) than individuals who consumed red meat < 2 times per week. Positive associations were also observed for intakes of processed meat (≥ 2 times per week vs. < 1 time/week, HR = 1.13, 95% CI = 1.05–1.21). The risk of CRC in individuals consuming alcohol ≥ 3 times/week was observed to be 8% higher than that in those consuming < 1 time/week (HR = 1.08, 95% CI = 1.01–1.17). In contrast, compared with individuals consuming < 200 mL/day of milk, those who consumed ≥ 300 mL/day had a 15% CRC risk reduction (HR = 0.85, 95% CI = 0.79–0.92). Compared with individuals who drank < 3 cups/day of tea, those who reported consumption ≥ 5 cups per day had a 12% lower risk of CRC (HR = 0.88, 95% CI = 0.82–0.94). Dose–response associations of red meat (p_trend_ < 0.001), milk (p_trend_ < 0.001), tea (p_trend_ < 0.001), and alcohol (p_trend_ = 0.01) consumption with CRC risk were observed.

**Table 2:** Dietary intakes and colorectal cancer risk in total study population

Dietary factor	No. cases	No. participants	Person-years	Crude HR (95% CI)	Adjusted HR (95% CI)
**WCRF dietary score**					
2–3	2,298	201,039	2,430,594	1.00 (ref.)	1.00 (ref.)
0–1	2,335	169,863	2,044,261	**1.27 (1.20–1.34)**	**1.12 (1.05–1.19)**
**Red meat (times/week)**					
< 2	2,086	184,189	2,225,060	1.00 (ref.)	1.00 (ref.)
2 to < 3	1,370	107,765	1,300,269	1.07 (1.00–1.15)	1.03 (0.97–1.11)
≥ 3	1,225	81,539	980,149	**1.24 (1.16–1.33)**	**1.16 (1.08–1.25)**
P-trend				** < 0.001**	** < 0.001**
**Processed meat (times/week)**					
< 1	1,567	141,439	1,716,522	1.00 (ref.)	1.00 (ref.)
1	1,423	112,069	1,352,197	**1.16 (1.08–1.24)**	1.06 (0.99–1.14)
≥ 2	1,690	119,967	1,436,538	**1.34 (1.25–1.43)**	**1.13 (1.05–1.21)**
P-trend				** < 0.001**	0.06
**Poultry (times/week)**					
< 1	703	57,209	687,085	1.00 (ref.)	1.00 (ref.)
1	1,833	136,573	1,643,714	1.06 (0.97–1.15)	1.05 (0.96–1.14)
≥ 2	2,142	179,627	2,173,735	1.02 (0.94–1.11)	1.02 (0.94–1.11)
P-trend				0.67	0.77
**Total fish (times/week)**					
≤ 1	1,104	96,791	1,168,954	1.00 (ref.)	1.00 (ref.)
> 1 to ≤ 2	2,272	172,168	2,077,449	1.02 (0.95–1.10)	1.05 (0.98–1.13)
> 2	1,303	104,601	1,259,732	0.93 (0.86–1.01)	0.96 (0.89–1.05)
P-trend				**0.02**	0.12
**Milk (100 mL/day)**					
< 2	1,640	125,072	1,510,035	1.00 (ref.)	1.00 (ref.)
2 to < 3	1,861	147,570	1,781,978	**0.89 (0.83–0.95)**	**0.91 (0.85–0.97)**
≥ 3	989	82,256	988,713	**0.85 (0.78–0.92)**	**0.85 (0.79–0.92)**
P-trend				** < 0.001**	** < 0.001**
**Cheese (times/week)**					
< 2	1,849	147,534	1,780,536	1.00 (ref.)	1.00 (ref.)
2 to 4	2,111	170,015	2,051,719	1.02 (0.96–1.08)	0.99 (0.93–1.06)
≥ 5	609	47,933	577,864	1.08 (0.98–1.18)	1.05 (0.95–1.15)
P-trend				0.14	0.46
**Total fruit (servings/day)**					
< 2	1,644	125,649	1,507,039	1.00 (ref.)	1.00 (ref.)
2 to < 4	2,038	168,670	2,040,211	**0.85 (0.80–0.91)**	0.93 (0.87–1.00)
≥ 4	998	79,126	957,664	**0.84 (0.77–0.90)**	0.95 (0.88–1.03)
P-trend				** < 0.001**	0.25
**Total vegetables (servings/day)**					
< 4	1,649	133,050	1,606,032	1.00 (ref.)	1.00 (ref.)
< 6	1,614	127,678	1,541,178	0.95 (0.89–1.02)	1.00 (0.93–1.07)
≥ 6	1,384	111,145	1,338,968	**0.93 (0.86–0.99)**	0.97 (0.90–1.04)
P-trend				**0.04**	0.37
**Coffee (cups/day)**					
< 1	1,258	104,995	1,268,205	1.00 (ref.)	1.00 (ref.)
1 to ≤ 2	1,805	144,409	1,742,423	0.96 (0.89–1.03)	0.96 (0.89–1.03)
> 2	1,614	123,992	1,493,625	1.05 (0.97–1.13)	1.01 (0.93–1.08)
P-trend				**0.04**	0.47
**Tea (cups/day)**					
< 3	1,898	147,056	1,775,758	1.00 (ref.)	1.00 (ref.)
< 5	1,413	109,475	1,322,518	0.93 (0.87–1.00)	0.95 (0.89–1.02)
≥ 5	1,367	116,770	1,404,889	**0.87 (0.81–0.93)**	**0.88 (0.82–0.94)**
P-trend				** < 0.001**	** < 0.001**
**Alcohol (times/week)**					
< 1	1,212	104,780	1,258,892	1.00 (ref.)	1.00 (ref.)
1 to 2	1,127	99,060	1,200,103	1.03 (0.95–1.12)	0.98 (0.90–1.06)
≥ 3	2,340	169,903	2,049,364	**1.18 (1.10–1.26)**	**1.08 (1.01–1.17)**
P-trend				** < 0.001**	**0.01**
**IV-weighted dietary score**					
-13.22 to < -2.88	1,248	115,317	1,393,664	1.00 (ref.)	1.00 (ref.)
-2.88 to < 1.09	1,449	115,642	1,397,328	**1.18 (1.10–1.28)**	**1.14 (1.06–1.23)**
1.09 to 13.02	1,656	115,412	1,389,266	**1.40 (1.30–1.50)**	**1.27 (1.18–1.37)**
P-trend				** < 0.001**	** < 0.001**

The HRs were estimated using Cox proportional hazard models with adjustment for sex, first-degree family history of colorectal cancer, smoking status, alcohol consumption (except when alcohol intake and dietary score are exposure), body mass index, and physical activity

*HR* hazard ratio, *CI* confidence interval, *WCRF* World Cancer Research Fund, *IV* inverse variance. Bold font indicates significant difference

In the stratification analysis by sex, unfavorable diets of both dietary scores and habitual intakes of red meat, processed meat, and alcohol were associated with an increased risk of CRC in men only. In addition, the inverse associations of the highest categories of milk and tea consumption with CRC risk occurred in both men and women (Additional file 1: Table S4).

In the subgroup analysis by cancer subsites, we found an increased risk of both colon and rectal cancer for those with unhealthy diets and high intakes of red and processed meats. Additionally, inverse associations of milk and tea consumption with colon cancer and fruit intake with rectal cancer were observed. Alcohol consumption was shown to be related to rectal but not colon cancer risks (Additional file 1: Table S5).

### Polygenic risk scores and associations with colorectal cancer

The association between the PRS and CRC risk is summarized in Additional file 1: Table S6. Compared to the lowest tertile, individuals in the highest tertile of the PRS had an increased risk of CRC by 98% (HR = 1.98, 95% CI = 1.84–2.13) for unweighted PRS, 109% (HR = 2.09, 95% CI = 1.94–2.24) for standardized weighted PRS, and 112% (HR = 2.12, 95% CI = 1.97–2.29) for IV-weighted PRS. In the subgroups, the highest tertile PRS exerted an approximately twofold higher risk of CRC incidence compared to the lowest tertile PRS.

**Fig. 2:**
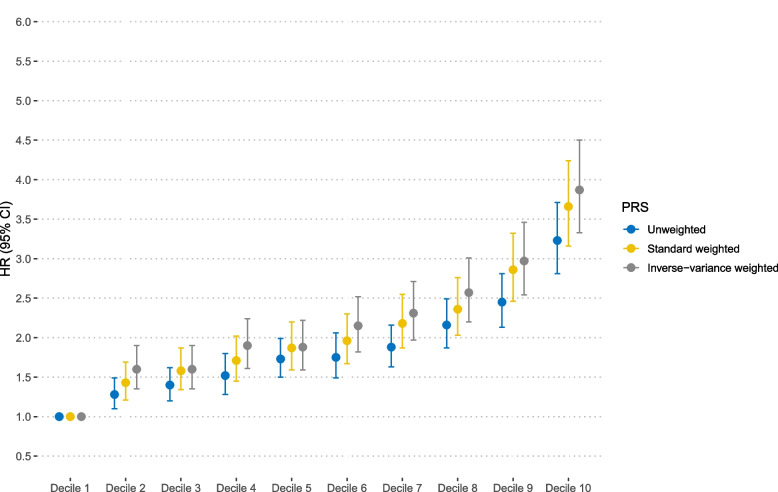
Hazard ratios and 95% confidence intervals for colorectal cancer risk by deciles of each polygenic risk score. The HRs were estimated using Cox proportional hazard models with adjustment for sex and first-degree family history of colorectal cancer. PRS, polygenic risk score; HR, hazard ratio; CI, confidence interval

All deciles showed significantly increased risks of CRC in a dose–response manner (p_trend_ < 0.001). The adjusted HRs (95% CIs) of the highest decile compared to the lowest decile were 3.23 (2.81–3.71) for unweighted PRS, 3.66 (3.16–4.24) for standardized weighted PRS, and 3.87 (3.33–4.50) for IV-weighted PRS. In the subgroups, the tenth decile PRS exerted an approximately three to fourfold higher risk of CRC incidence compared to the first decile PRS (Additional file 1: Table S6, Fig. 2; Additional file 2: Figures S1-S2).

### Interaction of diets and polygenic risk scores on CRC risk

**Fig. 3:**
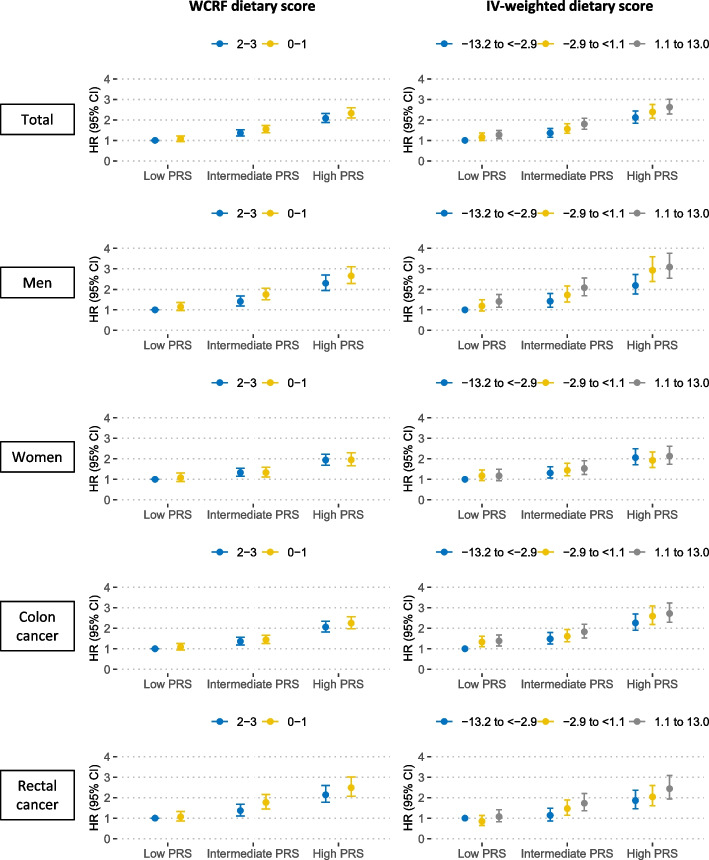
Overall and subgroup analyses by sex and subsites for joint effects of dietary and polygenic risk scores on colorectal cancer risk IV, inverse variance; PRS, polygenic risk score. Low PRS: 316 to < 454; intermediate PRS: 454 to < 483; high PRS: 483 to ≤ 621

Both dietary factors and the PRS were independently associated with CRC risk (p_interaction_ > 0.05) (Table 3). For the WCRF and IV-weighted dietary scores, participants with a high genetic risk had more than double the risk of developing CRC compared to those with a healthy dietary intake and a low genetic risk, regardless of the score category. Notably, the point estimates (HRs) tended to increase across dietary score categories within each PRS category and across PRS categories (Fig. 3). For all food groups and food items, more than double the risk of developing CRC was also observed in participants with unfavorable diets and a high genetic risk compared to those with a healthy dietary intake and a low genetic risk. The estimated joint associations were slightly higher in the men subgroup than in the women subgroup but were equivalent for the colon and rectal cancer subsites (Fig. 3 and Additional file 1: Tables S7-S8).

**Table 3:** Joint effects of dietary intake and polygenic risk score on colorectal cancer risk in total participants

Dietary factor	Low PRS			Intermediate PRS			High PRS			P_interaction_
	**No. cases**	**Person-years**	**HR (95% CI)**	**No. cases**	**Person-years**	**HR (95% CI)**	**No. cases**	**Person-years**	**HR (95% CI)**	
**Total population**										
**WCRF dietary score**										
2–3	519	812,923	1.00 (ref.)	707	810,979	1.36 (1.22–1.53)	1,072	806,692	2.08 (1.88–2.32)	0.83
0–1	509	681,901	1.08 (0.95–1.22)	729	681,155	1.55 (1.38–1.73)	1,097	681,205	2.33 (2.10–2.59)	
**Red meat (times/week)**										
< 2	462	748,267	1.00 (ref.)	635	739,638	1.39 (1.23–1.56)	989	737,155	2.19 (1.96–2.44)	0.83
2 to < 3	302	433,038	1.04 (0.90–1.20)	437	434,943	1.50 (1.31–1.71)	631	432,288	2.17 (1.93–2.45)	
≥ 3	274	324,027	1.19 (1.03–1.39)	382	327,432	1.65 (1.44–1.89)	569	328,690	2.44 (2.15–2.76)	
**Processed meat (times/week)**										
< 1	345	573,384	1.00 (ref.)	481	574,369	1.38 (1.21–1.59)	741	568,769	2.16 (1.90–2.45)	0.80
1	319	448,827	1.08 (0.93–1.26)	455	450,863	1.54 (1.34–1.77)	649	452,507	2.19 (1.92–2.50)	
≥ 2	373	482,937	1.12 (0.96–1.29)	517	476,784	1.57 (1.37–1.80)	800	476,817	2.43 (2.14–2.76)	
**Poultry (times/week)**										
≥ 2	463	721,709	1.00 (ref.)	676	728,130	1.45 (1.29–1.63)	1,003	723,896	2.16 (1.93–2.41)	0.77
1	419	551,619	1.07 (0.94–1.23)	569	545,482	1.47 (1.30–1.66)	845	546,613	2.19 (1.95–2.45)	
< 1	157	231,662	1.00 (0.83–1.19)	207	228,237	1.33 (1.13–1.57)	339	227,185	2.20 (1.91–2.54)	
**Total fish (times/week)**										
> 2	290	424,526	1.00 (ref.)	405	418,437	1.41 (1.21–1.64)	608	416,769	2.14 (1.86–2.46)	0.97
> 1 to ≤ 2	495	690,774	1.08 (0.93–1.25)	711	693,560	1.55 (1.35–1.77)	1,066	693,116	2.32 (2.04–2.65)	
≤ 1	252	390,096	1.08 (0.91–1.28)	337	390,221	1.43 (1.22–1.68)	515	388,637	2.20 (1.91–2.55)	
**Milk (100 mL/day)**										
≥ 3	214	330,834	1.00 (ref.)	293	328,379	1.37 (1.15–1.64)	482	329,500	2.25 (1.91–2.64)	0.51
2 to < 3	424	596,161	1.12 (0.95–1.31)	597	593,079	1.58 (1.35–1.85)	840	592,738	2.23 (1.92–2.59)	
< 2	361	503,725	1.19 (1.00–1.41)	510	504,997	1.68 (1.43–1.97)	769	501,314	2.56 (2.20–2.97)	
**Cheese (times/week)**										
< 2	397	594,359	1.00 (ref.)	558	593,345	1.40 (1.24–1.60)	894	592,832	2.26 (2.01–2.54)	0.21
2 to 4	493	685,943	1.08 (0.94–1.23)	670	682,791	1.47 (1.30–1.66)	948	682,984	2.08 (1.85–2.34)	
≥ 5	132	193,473	1.05 (0.87–1.28)	183	193,811	1.46 (1.22–1.74)	294	190,580	2.38 (2.05–2.77)	
**Total fruit (servings/day)**										
≥ 4	234	321,556	1.00 (ref.)	308	317,908	1.33 (1.12–1.58)	456	318,200	1.97 (1.68–2.31)	0.56
2 to < 4	450	682,784	0.93 (0.79–1.09)	617	681,033	1.27 (1.09–1.48)	971	676,394	2.03 (1.76–2.34)	
< 2	354	500,812	0.97 (0.83–1.15)	528	502,921	1.45 (1.24–1.70)	762	503,306	2.08 (1.80–2.42)	
**Total vegetables (servings/day)**										
≥ 6	332	450,423	1.00 (ref.)	386	445,940	1.18 (1.01–1.36)	666	442,604	2.05 (1.79–2.33)	0.05
4 to < 6	349	515,188	0.94 (0.81–1.09)	516	512,290	1.38 (1.21–1.59)	749	513,701	2.01 (1.77–2.29)	
< 4	350	533,182	0.93 (0.80–1.08)	540	537,844	1.42 (1.24–1.63)	759	535,006	2.00 (1.76–2.28)	
**Coffee (cups/day)**										
> 2	369	501,768	1.00 (ref.)	493	496,918	1.35 (1.18–1.54)	629	468,064	2.08 (1.83–2.35)	0.82
1 to ≤ 2	388	581,794	0.90 (0.78–1.04)	578	581,380	1.35 (1.18–1.53)	684	441,784	1.96 (1.73–2.22)	
< 1	281	421,501	0.99 (0.84–1.15)	383	423,437	1.33 (1.15–1.54)	873	587,633	2.07 (1.81–2.35)	
**Tea (cups/day)**										
≥ 5	322	467,205	1.00 (ref.)	416	469,620	1.28 (1.11–1.48)	752	494,939	1.93 (1.69–2.21)	0.32
3 to < 5	291	442,677	0.94 (0.80–1.10)	438	438,057	1.43 (1.24–1.65)	839	579,249	2.23 (1.95–2.54)	
< 3	425	594,604	1.07 (0.93–1.24)	600	593,522	1.51 (1.32–1.73)	594	423,267	2.24 (1.97–2.55)	
**Alcohol (times/week)**										
< 1	260	418,909	1.00 (ref.)	373	420,935	1.44 (1.22–1.68)	579	419,048	2.23 (1.93–2.58)	0.74
1 to 2	263	401,076	1.07 (0.90–1.27)	353	399,874	1.42 (1.21–1.67)	511	399,153	2.07 (1.78–2.40)	
≥ 3	515	686,270	1.11 (0.95–1.29)	727	682,105	1.57 (1.36–1.81)	1,098	680,988	2.38 (2.08–2.73)	
**IV-weighted dietary score**										
-13.22 to < -2.88	279	467,287	1.00 (ref.)	377	461,715	1.36 (1.16–1.59)	592	464,662	2.12 (1.84–2.44)	0.95
-2.88 to < 1.09	329	466,751	1.16 (0.99–1.36)	447	468,417	1.56 (1.35–1.81)	673	462,160	2.39 (2.08–2.75)	
1.09 to 13.02	370	463,047	1.27 (1.08–1.48)	523	462,858	1.80 (1.55–2.08)	763	463,360	2.62 (2.29–3.01)	

Low PRS: 316 to < 454; intermediate PRS: 454 to < 483; high PRS: 483 to ≤ 621. The HRs were estimated using Cox proportional hazard models with adjustment for sex (except for sex-specific analyses), first-degree family history of colorectal cancer, household income, smoking status, alcohol consumption (except when alcohol intake and dietary score are exposures), body mass index, and physical activity

*PRS* polygenic risk score, *HR* hazard ratio, *CI* confidence interval, *WCRF* World Cancer Research Fund, *IV* inverse variance

### Associations between dietary intake and CRC risk according to PRS tertiles

**Fig. 4:**
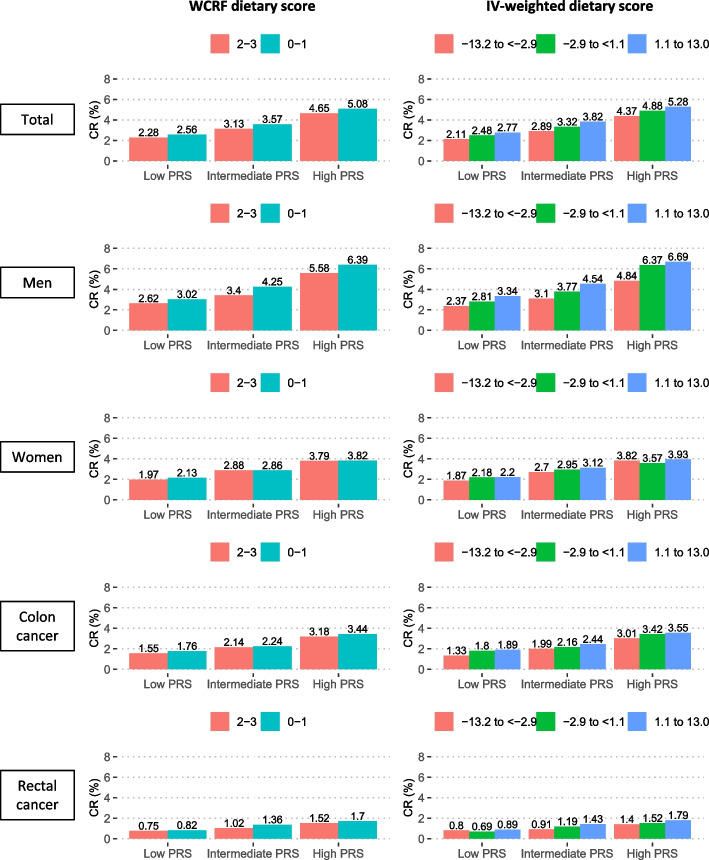
Estimates of cumulative risk of developing colorectal cancer at age 80 years according to dietary and polygenic risk score categories CR, cumulative risk; WCRF, World Cancer Research Fund; IV, inverse variance; PRS, polygenic risk score. Low PRS: 316 to < 454; intermediate PRS: 454 to < 483; high PRS: 483 to ≤ 621

Of participants with a high genetic risk and an unfavorable diet based on the WCRF dietary score, the cumulative risk of CRC at the age of 80 years was estimated to be 5.08% vs. 2.28% in participants with a low genetic risk and a high adherence to WCRF/AICR dietary recommendations. Of participants with a high genetic risk and an unfavorable diet based on the IV-weighted dietary score, the cumulative risk of CRC at the age of 80 years was estimated to be 5.28% vs. 2.11% in participants with a low genetic risk and a healthy dietary habit (Fig. 4 and Table 4). Subgroup analyses showed a higher cumulative risk in men than in women and for colon cancer and rectal cancer (Fig. 4 and Additional file 1: Tables S9-S10).

**Table 4:** Estimates of cumulative risk of developing colorectal cancer at age 80 years and associations between dietary intake and colorectal cancer according to polygenic risk score categories in total participants

Dietary factor	Low PRS		Intermediate PRS		High PRS	
	**CR (%)**	**HR (95% CI)**	**CR (%)**	**HR (95% CI)**	**CR (%)**	**HR (95% CI)**
**WCRF dietary score**						
2–3	2.28	1.00 (ref.)	3.13	1.00 (ref.)	4.65	1.00 (ref.)
0–1	2.56	1.12 (0.99–1.28)	3.57	**1.14 (1.02–1.27)**	5.08	1.09 (1.00–1.19)
**Red meat (times/week)**						
< 2	2.27	1.00 (ref.)	3.14	1.00 (ref.)	4.74	1.00 (ref.)
2 to < 3	2.37	1.05 (0.90–1.21)	3.38	1.08 (0.95–1.22)	4.71	0.99 (0.90–1.10)
≥ 3	2.73	**1.20 (1.04–1.40)**	3.72	**1.19 (1.04–1.35)**	5.26	1.11 (1.00–1.24)
**Processed meat (times/week)**						
< 1	2.22	1.00 (ref.)	3.10	1.00 (ref.)	4.70	1.00 (ref.)
1	2.45	1.11 (0.95–1.29)	3.45	1.11 (0.98–1.27)	4.71	1.00 (0.90–1.12)
≥ 2	2.58	1.17 (1.00–1.36)	3.52	1.14 (1.00–1.29)	5.15	1.10 (0.99–1.22)
**Poultry (times/week)**						
< 1	2.33	1.00 (ref.)	3.07	1.00 (ref.)	4.95	1.00 (ref.)
1	2.51	1.08 (0.90–1.30)	3.4	1.11 (0.94–1.30)	4.90	0.99 (0.87–1.12)
≥ 2	2.35	1.01 (0.84–1.21)	3.39	1.10 (0.94–1.29)	4.79	0.97 (0.85–1.09)
**Total fish (times/week)**						
≤ 1	2.48	1.00 (ref.)	3.27	1.00 (ref.)	4.73	1.00 (ref.)
> 1 to ≤ 2	2.46	0.99 (0.85–1.15)	3.49	1.07 (0.94–1.22)	5.04	1.07 (0.96–1.19)
> 2	2.27	0.91 (0.77–1.08)	3.18	0.97 (0.84–1.13)	4.66	0.99 (0.87–1.11)
**Milk (100 mL/day)**						
< 2	2.60	1.00 (ref.)	3.64	1.00 (ref.)	5.26	1.00 (ref.)
2 to < 3	2.43	0.93 (0.81–1.08)	3.40	0.94 (0.83–1.05)	4.64	**0.88 (0.80–0.97)**
≥ 3	2.19	0.84 (0.71–1.00)	2.96	**0.81 (0.70–0.94)**	4.67	**0.89 (0.79–0.99)**
**Cheese (times/week)**						
< 2	2.31	1.00 (ref.)	3.25	1.00 (ref.)	5.02	1.00 (ref.)
2 to 4	2.52	1.09 (0.95–1.25)	3.39	1.04 (0.93–1.17)	4.60	0.92 (0.84–1.00)
≥ 5	2.47	1.07 (0.88–1.31)	3.36	1.03 (0.87–1.22)	5.23	1.05 (0.92–1.20)
**Total fruit (servings/day)**						
< 2	2.47	1.00 (ref.)	3.62	1.00 (ref.)	4.94	1.00 (ref.)
2 to < 4	2.32	0.94 (0.81–1.08)	3.16	**0.87 (0.77–0.98)**	4.85	0.98 (0.89–1.08)
≥ 4	2.49	1.01 (0.85–1.19)	3.29	0.91 (0.79–1.05)	4.74	0.96 (0.85–1.08)
**Total vegetables (servings/day)**						
< 4	2.35	1.00 (ref.)	3.55	1.00 (ref.)	4.78	1.00 (ref.)
4 to < 6	2.36	1.00 (0.86–1.17)	3.45	0.97 (0.86–1.10)	4.85	1.01 (0.92–1.12)
≥ 6	2.52	1.07 (0.92–1.25)	2.94	**0.83 (0.72–0.94)**	4.92	1.03 (0.93–1.14)
**Coffee (cups/day)**						
< 1	2.48	1.00 (ref.)	3.33	1.00 (ref.)	4.92	1.00 (ref.)
1 to ≤ 2	2.26	0.91 (0.78–1.06)	3.35	1.00 (0.88–1.14)	4.72	0.96 (0.86–1.06)
> 2	2.53	1.02 (0.87–1.19)	3.36	1.01 (0.88–1.16)	4.95	1.00 (0.90–1.12)
**Tea (cups/day)**						
< 3	2.56	1.00 (ref.)	3.59	1.00 (ref.)	5.05	1.00 (ref.)
3 to < 5	2.24	0.87 (0.75–1.01)	3.37	0.94 (0.83–1.06)	5.07	1.00 (0.91–1.11)
≥ 5	2.39	0.93 (0.81–1.08)	3.03	**0.84 (0.74–0.96)**	4.40	**0.87 (0.78–0.96)**
**Alcohol (times/week)**						
< 1	2.19	1.00 (ref.)	3.22	1.00 (ref.)	4.87	1.00 (ref.)
1 to 2	2.41	1.10 (0.93–1.31)	3.21	1.00 (0.86–1.15)	4.45	0.91 (0.81–1.03)
≥ 3	2.55	1.17 (1.00–1.37)	3.51	1.09 (0.92–1.25)	5.08	1.04 (0.94–1.16)
**IV-weighted dietary score**						
-13.22 to < -2.88	2.11	1.00 (ref.)	2.89	1.00 (ref.)	4.37	1.00 (ref.)
-2.88 to < 1.09	2.48	1.17 (1.00–1.38)	3.32	**1.15 (1.01–1.32)**	4.88	1.12 (1.00–1.25)
1.09 to 13.02	2.77	**1.32 (1.12–1.54)**	3.82	**1.34 (1.17–1.53)**	5.28	**1.21 (1.08–1.35)**

Low PRS: 316 to < 454; intermediate PRS: 454 to < 483; high PRS: 483 to ≤ 621. The HRs were estimated using Cox proportional hazard models with adjustment for sex, first-degree family history of colorectal cancer, household income, smoking status, alcohol consumption (except when alcohol intake and dietary score are exposures), body mass index, and physical activity

*PRS* polygenic risk score, *CR* cumulative risk, *HR* hazard ratio, *CI* confidence interval, *WCRF* World Cancer Research Fund, *IV* inverse variance

In further stratification analyses by PRS categories, we found that a low score of the WCRF diet was significantly associated with CRC in individuals with intermediate genetic risk (HR = 1.14, 95% CI = 1.02–1.27), whereas the estimates in low (HR = 1.12, 95% CI = 0.99–1.28) and high (HR = 1.09, 95% CI = 1.00–1.19) genetic risk groups were marginal. In contrast, we found that a high score on the IV-weighted diet was significantly associated with CRC across genetic risk groups, with HRs (95% CIs) of 1.32 (1.12–1.54), 1.34 (1.17–1.53), and 1.21 (1.08–1.35) for individuals with low, intermediate, and high PRS, respectively (Table 4). Similar findings were observed in men, colon cancer, and rectal cancer subgroup analyses for the IV-weighted dietary score but only in the men subgroup for the WCRF dietary score (Additional file 1: Tables S9-S10). Less adherence to the WCRF/AICR dietary recommendation exerted an increased risk of CRC in men with high genetic risk (Additional file 1: Table S9).

Figure 4 presents the cumulative risk of CRC at the age of 80 years for individuals defined jointly by dietary scores and PRS categories. The excess risks were 0.28% (2.56% vs. 2.28%) due to the WCRF diet alone and 1.37% (4.65% vs. 2.28%) due to the PRS. The excess risks were 0.66% (2.77% vs. 2.11%) due to the IV-weighted diet alone and 2.26% (4.37% vs. 2.11%) due to the PRS. Compared to dietary factors, the risk of CRC was more likely attributable to genetic factors in the analysis of the overall study population and subgroup analyses by sex and CRC subsites (Additional file 1: Table S11).

## Discussion

In this large prospective study to investigate how dietary intake and genetic risk contribute to the risk of CRC, we found that both an unhealthy diet and increased genetic risk were independently associated with the incidence of CRC without evidence of significant interactions. Regarding dietary components, we found that UK Biobank participants with frequent consumption of red meat, processed meat, or alcohol had an increased risk of CRC, whereas those who commonly drank milk and tea had a decreased risk of CRC. In individuals with high genetic risks, adherence to a healthy dietary habit of 11 foods overall but not the WCRF dietary recommendations alone may be beneficial in CRC risk reductions. Overall, CRC risk was more attributed to increased genetic risk than unhealthy dietary habit.

Evidence from systematic reviews for the association between dietary intake and CRC risk was available in the WCRF Continuous Update Project [34]. Our estimates were similar to those reported from the WCRF Continuous Update Project, with a 12% increased risk of CRC for red meat (relative risk per 100 g/day = 1.12, 95% CI = 1.00–1.25) and an 18% increased risk of CRC for processed meat (relative risk per 100 g/day = 1.18, 95% CI = 1.10–1.28) intake [34]. An umbrella review for updated evidence consistently found positive associations between red meat and processed meat intake and CRC risk [35]. It has been hypothesized that DNA damage is caused by carcinogenic substances, such as heme iron, *N*-nitroso-compounds, heterocyclic aromatic amines, and polycyclic aromatic hydrocarbons, especially when processing meat or cooking at high temperature [36, 37]. The International Agency for Research on Cancer further emphasized the roles of some heavy metals and other persistent organic contaminants in raw or unprocessed meat, which also indicates the carcinogenicity of red and processed meat consumption [38].

CRC risks can differ between men and women due to endocrine differences, which modulate gene expression after dimerization and translocation to the nucleus [39, 40]. Estrogen exhibits its anti-CRC effects through estrogen receptor (ER) superfamilies, including ER-α and ER-β [39, 40]. While the expression of ER-α is low in both colon cancer and normal colonic cells, there is a significant decrease in ER-β in colonic neoplasms compared to normal colonic mucosa, which results in hyperproliferation, reduced differentiation, and anti-apoptosis [40]. This ER-β reduction was also significantly lower in women than in men [41]. In the present study, the associations of red and/or processed meat with CRC risks appeared to be slightly stronger in men than in women, which might be explained by a lower risk in women than in men due to less meat consumption (mean in men and women: 2.3 and 2.0 times/week for red meat and 2.2 and 1.6 times/week for processed meat, respectively; *p* < 0.001). In addition, the carcinogenic effect of heme irons in meat-rich diets could be weakened in women because of blood loss during menstruation [42, 43]. Overall, we observed a higher attributable fraction of CRC due to dietary intake in men than in women, which suggested that men may obtain more CRC risk reduction benefits than women by adhering to healthy dietary habits.

The preventive effect of tea consumption was attributed to its active ingredients, such as polyphenols, pigments, polysaccharides, alkaloids, free amino acids, and saponins [44]. Among them, the main active ingredient, epigallocatechin-3-gallate, is mainly absorbed in the intestine and metabolized by the gut microbiome and exerts antioxidation, growth inhibition, and apoptosis induction effects [45]. Evidence in human colon cancer cells supports the bioactivity of epigenetic modifications against colon cancer [45]. Furthermore, the results from a recent meta-analysis of 20 prospective cohort studies showed a similar direction of the association between tea intake and CRC (pooled relative risk 0.97, 95% CI = 0.94–1.01) [46]. However, our study is still limited in assessing several factors that may confer the estimates, such as tea temperature and concentration, age at initiation of drinking, and drinking duration.

In general, the impact of dietary factors on CRC incidence observed in our study is in line with previous reports by Bradbury et al. [23]. However, the present study has extended the investigations of the previous study in several ways. First, we derived both the WCRF and IV-weighted dietary scores to obtain robust findings. A previous study created an a priori dietary score from seven dietary factors and examined its interaction with the genetic risk of upper gastrointestinal cancer [47]. However, dietary components were selected based on recommendations on cardiometabolic health [47]. Another study developed a healthy lifestyle score based on the WCRF/AICR and American Cancer Society guidelines on cancer prevention [24]. Adopting a similar approach of limiting to dietary factors (red and processed meat, fruit and vegetables, and alcohol) only, we did not detect any interactions with the PRS on CRC risk in the present study. We further extended the dietary score calculation by weighting all 11 dietary factors (red meat, processed meat, poultry, fish, milk, cheese, fruit, vegetables, coffee, tea, and alcohol), which were reported by the WCRF/AICR for their effects on CRC risk; however, we still confirmed the independence between dietary and genetic factors on CRC incidence.

Second, we derived variants of CRC susceptibility from largely up-to-date meta-analyses rather than a single GWAS. Previous studies systematically reviewed and used UK Biobank data to externally validate the predictive performance of the PRS in which CRC susceptibility loci were identified from 14 GWASs [48, 49]. The number of SNPs was 120 in Huyghen’s study and from 6 to 63 in other studies, and the AUC of the genetics-plus-age-plus-family history model ranged between 0.65 and 0.68 in men and between 0.61 and 0.65 in women [48, 49]. However, the highest AUC was obtained from the model, including SNPs identified in the Huyghen’ study, which overlapped with UK Biobank data [48]. Our present study determined an overall AUC of 0.60–0.61 according to different weighted approaches, and the estimates from independent datasets that were not taken from UK Biobank can minimize any substantial inflations due to overlap between the base and target data and provide an unbiased evaluation for the developed model. By upweighting the effect of risk alleles, the PRS estimated from the IV-weighted approach showed a greater dose–response association with the CRC risk than that from the unweighted and standard-weighted approach.

Third, by approaching the gene-environment interaction framework, we included the PRS to better understand the role of dietary intake in CRC risk. The precise pathways for the joint effect of dietary intake and the PRS in CRC risk remain unclear. This might be proposed by the natural pleiotropic effect of each factor in overlapping with pathways of CRC development. To explore possible biological mechanisms, we performed a gene-set enrichment analysis of 98 SNPs included in the PRS to identify functional pathways of these variants using a web-based FUMA tool [50]. Accordingly, CRC susceptibility loci included in the PRS were mainly involved in the metabolism of PUFAs (Additional file 2: Figure S3). This result supported findings from a previous study on the link between genetically predicted PUFAs and CRC risk [17] and thus suggested a possible interaction between dietary intake and the PRS for CRC via PUFA metabolism pathways. A large-scale study from the Genetics and Epidemiology of Colorectal Cancer Consortium and Colon Cancer Family Registry examined the effect modification of candidate CRC susceptibility on selected dietary factors, such as red meat, processed meat, fruit, vegetables, and fiber [51]. However, none of the significant interactions was detected after accounting for multiple comparisons [51]. Our findings of a greater number of SNPs in the PRS also suggested no evidence of gene-diet interactions in the risk of CRC.

There are also some limitations that need to be addressed. First, the FFQ was used to quantify information on an individual’s intake during the preceding year. There could be difficulty in recalling some food items accurately. Second, our findings require validation in populations of ethnic backgrounds other than White British.

In summary, the current study provided evidence that unhealthy dietary intake and genetic risk were independently associated with the risk of CRC. Adherence to a healthy dietary habit regarding the above 11 foods may attenuate CRC incidence in individuals at high genetic risk.

### Supplementary Information

**Additional file 1.** 

**Additional file 2.**

## Data Availability

The UK Biobank is an open access resource, available at https://www.ukbiobank.ac.uk/researchers/, and can be obtained from the UK Biobank by submitting a data request proposal. The data that support findings of this study were used under license for the current study (Application #94695), and so are not publicly available.

## Abbreviations

AICR: American Institute for Cancer Research
BMI: Body mass index
CRC: Colorectal cancer
ER: Estrogen receptor
GWAS: Genome-wide association study
HR: Hazard ratio
IV: Inverse variance
LD: Linkage disequilibrium
PRS: Polygenic risk score
PUFA: Polyunsaturated fatty acids
SE: Standard error
SNP: Single nucleotide polymorphism
WCRF: World Cancer Research Fund
