# Supplementary material for: Assessments of dietary intake and polygenic risk score in associations with colorectal cancer risk: evidence from the UK Biobank
Source: BMC Cancer. 2023 Oct 18;23:993. doi: 10.1186/s12885-023-11482-1 (PMC10583398; doi:10.1186/s12885-023-11482-1)
Supplement: Supplementary file 1 — Additional file 1. [file 12885_2023_11482_MOESM1_ESM.docx]

**Assessments of dietary intake and polygenic risk score in associations with colorectal cancer risk: evidence from the UK Biobank**

**Additional file 1: Supplementary Tables**

**Table S1.** UK Biobank touch-screen Questionnaire of food intake in the UK Biobank

| **Food item** | **Question stem** | **Response** | **Validation** | **Hints** |
| --- | --- | --- | --- | --- |
| **Oily fish** | How often do you eat oily fish? (e.g. sardines, salmon, mackerel, herring) | SELECT one of 8 from 0:Never 1: Less than once a week 2: Once a week 3: 2-4 times a week 4: 5-6 times a week 5: Once or more daily -1: Do not know -3: Prefer not to answer |  | Please provide an average considering your intake over the last year. If you are unsure, please provide an estimate or select Do not know. Oily fish include: Salmon, Anchovies, Trout, Swordfish, Mackerel, Bloater, Herring, Cacha, Sardines, Carp, Pilchards, Hilsa, Kipper, Jack fish, Eel, Katla,Whitebait, Orange roughy, Tuna (fresh only), Pangas, Sprats. |
| **Non-oily fish** | How often do you eat other types of fish? (e.g. cod, tinned tuna, haddock) | SELECT one of 8 from 0:Never 1: Less than once a week 2: Once a week 3: 2-4 times a week 4: 5-6 times a week 5: Once or more daily -1: Do not know -3: Prefer not to answer |  | Please provide an average considering your intake over the last year. If you are unsure, please provide an estimate or select Do not know. |
| **Processed meat** | How often do you eat processed meats? (such as bacon, ham, sausages, meat pies, kebabs, burgers, chicken nuggets) | SELECT one of 8 from 0:Never 1: Less than once a week 2: Once a week 3: 2-4 times a week 4: 5-6 times a week 5: Once or more daily -1: Do not know -3: Prefer not to answer |  | Please provide an average considering your intake over the last year If you are unsure, please provide an estimate or select Do not know. |
| **Beef** | How often do you eat beef? (Do not count processed meats) | SELECT one of 8 from 0:Never 1: Less than once a week 2: Once a week 3: 2-4 times a week 4: 5-6 times a week 5: Once or more daily -1: Do not know -3: Prefer not to answer |  | Please provide an average considering your intake over the last year If you are unsure, please provide an estimate or select Do not know. |
| **Lamb/mutton** | How often do you eat lamb/mutton? (Do not count processed meats) | SELECT one of 8 from 0:Never 1: Less than once a week 2: Once a week 3: 2-4 times a week 4: 5-6 times a week 5: Once or more daily -1: Do not know -3: Prefer not to answer |  | Please provide an average considering your intake over the last year If you are unsure, please provide an estimate or select Do not know. |
| **Pork** | How often do you eat pork? (Do not count processed meats such as bacon or ham) | SELECT one of 8 from 0:Never 1: Less than once a week 2: Once a week 3: 2-4 times a week 4: 5-6 times a week 5: Once or more daily -1: Do not know -3: Prefer not to answer |  | Please provide an average considering your intake over the last year If you are unsure, please provide an estimate or select Do not know. |
| **Poultry** | How often do you eat chicken, turkey or other poultry? (Do not count processed meats) | SELECT one of 8 from 0:Never 1: Less than once a week 2: Once a week 3: 2-4 times a week 4: 5-6 times a week 5: Once or more daily -1: Do not know -3: Prefer not to answer |  | Please provide an average considering your intake over the last year If you are unsure, please provide an estimate or select Do not know. |
| **Cheese** | How often do you eat cheese? (Include cheese in pizzas, quiches, cheese sauce etc) | SELECT one of 8 from 0:Never 1: Less than once a week 2: Once a week 3: 2-4 times a week 4: 5-6 times a week 5: Once or more daily -1: Do not know -3: Prefer not to answer |  | Please provide an average considering your intake over the last year If you are unsure, please provide an estimate or select Do not know. |
| **Cooked vegetables** | On average how many heaped tablespoons of COOKED vegetables would you eat per DAY? (Do not include potatoes; put '0' if you do not eat any) | Enter INTEGER OR -10: Less than one OR -1: Do not know OR -3: Prefer not to answer | Require: ≤ 50  Expect: undefined  Units: tablespoons | Please provide an average considering your intake over the last year. If you are unsure, please provide an estimate or select Do not know. If you have less than one tablespoon a day select Less than one. |
| **Salad/ raw vegetables** | On average how many heaped tablespoons of SALAD or RAW vegetables would you eat per DAY? (Include lettuce, tomato in sandwiches; put '0' if you do not eat any) | Enter INTEGER OR -10: Less than one OR -1: Do not know OR -3: Prefer not to answer | Require: ≤ 50  Expect: undefined  Units: tablespoons | Please provide an average considering your intake over the last year. If you are unsure, please provide an estimate or select Do not know. If you have less than one tablespoon a day select Less than one. |
| **Fresh fruit** | About how many pieces of FRESH fruit would you eat per DAY? (Count one apple, one banana, 10 grapes etc as one piece; put '0' if you do not eat any) | Enter INTEGER OR -10: Less than one OR -1: Do not know OR -3: Prefer not to answer | Require: ≤ 50  Expect: undefined  Units: pieces | Please provide an average considering your intake over the last year. If you are unsure, please provide an estimate or select Do not know. |
| **Dried fruit** | About how many pieces of DRIED fruit would you eat per DAY? (Count one prune, one dried apricot, 10 raisins as one piece; put '0' if you do not eat any) | Enter INTEGER OR -10: Less than one OR -1: Do not know OR -3: Prefer not to answer | Require: ≤ 100  Expect: undefined  Units: pieces | Please provide an average considering your intake over the last year. If you are unsure, please provide an estimate or select Do not know. |
| **Bread intake** | How many slices of bread do you eat each WEEK? | Enter INTEGER OR -10: Less than one OR -1: Do not know OR -3: Prefer not to answer | Require: ≥ 0, ≤ 250 Expect: ≤ 50  Units: slices | For other types of bread: - one bread roll = 2 slices  - one pitta bread = 2 slices |
| **Bread type** | What type of bread do you mainly eat? | SELECT one of 6 from  1: White  2: Brown  3: Wholemeal or wholegrain  4: Other type of bread  -1: Do not know  -3: Prefer not to answer |  | If you eat more than one type of bread, please select the one that you eat the most  If you are unsure, select Do not know |
| **Cereal intake** | How many bowls of cereal do you eat a WEEK? | Enter INTEGER OR -10: Less than one OR -1: Do not know OR -3: Prefer not to answer | Require: ≥ 0, ≤ 99 Expect: ≤ 14  Units: bowls | Please provide an average considering your intake over the last year. If you are unsure, please provide an estimate or select Do not know. |
| **Cereal type** | What type of cereal do you mainly eat? | SELECT one of 7 from  1: Bran cereal (e.g. All Bran, Branflakes)  2: Biscuit cereal (e.g. Weetabix)  3: Oat cereal (e.g. Ready Brek, porridge)  4: Mueli  5: Other (e.g. Cornflakes, Frosties)  -1: Do not know  -3: Prefer not to answer |  | If you eat more than one type of cereal, please select the one that you eat the most  If you are unsure, select Do not know |
| **Coffee** | How many cups of coffee do you drink each DAY? (Include decaffeinated coffee) | Enter INTEGER OR -10: Less than one OR -1: Do not know OR -3: Prefer not to answer | Require: ≥ 0, ≤ 99 Expect: ≤ 10  Units: cups | Please provide an average considering your intake over the last year. If you are unsure, please provide an estimate or select Do not know. |
| **Tea** | How many cups of tea do you drink each DAY? (Include black and green tea) | Enter INTEGER OR -10: Less than one OR -1: Do not know OR -3: Prefer not to answer | Require: ≥ 0, ≤ 99 Expect: ≤ 20  Units: cups | Please provide an average considering your intake over the last year. If you are unsure, please provide an estimate or select Do not know. |
| **Alcohol** | About how often do you drink alcohol? | SELECT one of 5 from  1: Daily or almost daily  2: Three or four times a week  3: Once or twice a week  4: One to three times a month  5: Special occasions only  6: Never  -3: Prefer not to answer |  | If this varies a lot, please provide an average considering your intake over the last year. |

**Table S2.** List of 98 variants for polygenic risk score calculation

| **Reference variant** | **Effect allele** | **Other allele** | **EAF** | **UK Biobank variant** | **Reference allele** | **Alternative allele** | **Correlated alleles** | **R^2^** |
| --- | --- | --- | --- | --- | --- | --- | --- | --- |
| **Available in UK Biobank** | | | | | | | | |
| rs72647484 | T | C | 0.91 | rs72647484 | C | T |  |  |
| rs6678517 | A | G | 0.59 | rs6678517 | G | A |  |  |
| rs17011141 | G | A | 0.21 | rs17011141 | G | A |  |  |
| rs11692435 | G | A | 0.90 | rs11692435 | A | G |  |  |
| rs75610640 | C | T | 0.16 | rs75610640 | C | T |  |  |
| rs11884596 | C | T | 0.39 | rs11884596 | C | T |  |  |
| rs983402 | T | C | 0.30 | rs983402 | C | T |  |  |
| rs13020391 | C | T | 0.63 | rs13020391 | T | C |  |  |
| rs35470271 | G | A | 0.15 | rs35470271 | G | A |  |  |
| rs9831861 | G | T | 0.59 | rs9831861 | G | T |  |  |
| rs6781752 | A | G | 0.21 | rs6781752 | A | G |  |  |
| rs35446936 | G | A | 0.70 | rs35446936 | A | G |  |  |
| rs13149359 | A | C | 0.37 | rs13149359 | A | C |  |  |
| rs1391441 | A | G | 0.65 | rs1391441 | A | G |  |  |
| rs75686861 | A | G | 0.10 | rs75686861 | A | G |  |  |
| rs77776598 | C | T | 0.06 | rs77776598 | C | T |  |  |
| rs2735940 | G | A | 0.5 | rs2735940 | G | A |  |  |
| rs7708610 | A | G | 0.35 | rs7708610 | A | G |  |  |
| rs12514517 | A | G | 0.29 | rs12514517 | A | G |  |  |
| rs12522693 | G | A | 0.85 | rs12522693 | A | G |  |  |
| rs2516420 | C | T | 0.93 | rs2516420 | T | C |  |  |
| rs16878812 | A | G | 0.89 | rs16878812 | G | A |  |  |
| rs6933790 | T | C | 0.83 | rs6933790 | C | T |  |  |
| rs62404966 | C | T | 0.76 | rs62404966 | T | C |  |  |
| rs12672022 | T | C | 0.84 | rs12672022 | C | T |  |  |
| rs10951878 | C | T | 0.49 | rs10951878 | T | C |  |  |
| rs3801081 | G | A | 0.68 | rs3801081 | G | A |  |  |
| rs3133285 | G | C | 0.82 | rs3133285 | C | G |  |  |
| rs16892766 | C | A | 0.09 | rs16892766 | C | A |  |  |
| rs4313119 | G | T | 0.75 | rs4313119 | T | G |  |  |
| rs34405347 | T | G | 0.90 | rs34405347 | G | T |  |  |
| rs10980628 | C | T | 0.21 | rs10980628 | C | T |  |  |
| rs7894531 | G | A | 0.69 | rs7894531 | A | G |  |  |
| rs10821907 | C | T | 0.83 | rs10821907 | T | C |  |  |
| rs704017 | G | A | 0.58 | rs704017 | G | A |  |  |
| rs2193352 | G | A | 0.19 | rs2193352 | G | A |  |  |
| rs12246635 | C | T | 0.1 | rs12246635 | C | T |  |  |
| rs4450168 | C | A | 0.17 | rs4450168 | C | A |  |  |
| rs4944940 | G | A | 0.96 | rs4944940 | A | G |  |  |
| rs2186607 | T | A | 0.50 | rs2186607 | A | T |  |  |
| rs3087967 | T | C | 0.30 | rs3087967 | C | T |  |  |
| rs35808169 | C | T | 0.17 | rs35808169 | C | T |  |  |
| rs10849438 | G | T | 0.12 | rs10849438 | G | T |  |  |
| rs11610543 | G | A | 0.52 | rs11610543 | G | A |  |  |
| rs12372718 | G | A | 0.39 | rs12372718 | G | A |  |  |
| rs4759277 | A | C | 0.33 | rs4759277 | C | T |  |  |
| rs7300312 | C | T | 0.57 | rs7300312 | C | T |  |  |
| rs10161980 | C | G | 0.62 | rs10161980 | G | C |  |  |
| rs7333607 | G | A | 0.22 | rs7333607 | G | A |  |  |
| rs78341008 | C | T | 0.07 | rs78341008 | C | T |  |  |
| rs1330889 | C | T | 0.87 | rs1330889 | C | T |  |  |
| rs17094983 | G | A | 0.88 | rs17094983 | A | G |  |  |
| rs17816465 | A | G | 0.20 | rs17816465 | A | G |  |  |
| rs4776316 | A | G | 0.73 | rs4776316 | G | A |  |  |
| rs7495132 | T | C | 0.12 | rs7495132 | T | C |  |  |
| rs143635270 | G | A | 0.99 | rs143635270 | A | G |  |  |
| rs9924886 | A | C | 0.73 | rs9924886 | C | A |  |  |
| rs61336918 | A | T | 0.29 | rs61336918 | T | A |  |  |
| rs12149163 | T | C | 0.50 | rs12149163 | C | T |  |  |
| rs899244 | T | C | 0.21 | rs899244 | T | C |  |  |
| rs4968127 | G | A | 0.37 | rs4968127 | A | G |  |  |
| rs73975588 | A | C | 0.87 | rs73975588 | C | A |  |  |
| rs17836917 | G | A | 0.98 | rs17836917 | A | G |  |  |
| rs983318 | A | G | 0.24 | rs983318 | A | G |  |  |
| rs75954926 | G | A | 0.64 | rs75954926 | G | A |  |  |
| rs11874392 | A | T | 0.55 | rs11874392 | T | A |  |  |
| rs34797592 | T | C | 0.11 | rs34797592 | T | C |  |  |
| rs73068325 | T | C | 0.18 | rs73068325 | T | C |  |  |
| rs189583 | G | C | 0.33 | rs189583 | C | G |  |  |
| rs994308 | C | T | 0.58 | rs994308 | T | C |  |  |
| rs28488 | T | C | 0.67 | rs28488 | T | C |  |  |
| rs11087784 | G | A | 0.15 | rs11087784 | G | A |  |  |
| rs2295444 | C | T | 0.50 | rs2295444 | T | C |  |  |
| rs6065668 | C | T | 0.74 | rs6065668 | T | C |  |  |
| rs2179593 | A | C | 0.72 | rs2179593 | A | C |  |  |
| rs4811050 | A | G | 0.18 | rs4811050 | A | G |  |  |
| rs6063514 | C | T | 0.61 | rs6063514 | T | C |  |  |
| rs3787089 | C | T | 0.32 | rs3787089 | T | C |  |  |
| **Not available in UK Biobank** | | | | | | | | |
| rs61776719 | C | A | 0.45 | rs28428561 | A | G | C=A, A=G | 0.81 |
| rs12144319 | C | T | 0.29 | rs628667 | C | T | T=T, C=C | 0.96 |
| rs812481 | G | C | 0.52 | rs1283532 | T | C | C=C, G=T | 0.95 |
| rs72942485 | G | A | 0.98 | rs76502974 | A | T | G=T, A=A | 1.00 |
| rs17035289 | T | C | 0.83 | rs57251748 | CT | C | T=-, C=T | 0.84 |
| rs3987 | G | A | 0.38 | rs4353970 | G | A | A=A, G=G | 0.86 |
| rs9271695 | G | A | 0.81 | rs9271513 | G | C | A=C, G=G | 0.96 |
| rs1321310 | C | T | 0.24 | rs9470361 | A | G | T=G, C=A | 0.98 |
| rs3125049 | G | A | 0.85 | rs6911381 | T | C | G=C, A=T | 0.97 |
| rs1412834 | T | C | 0.50 | rs2383206 | G | A | T=A, C=G | 0.98 |
| rs174533 | G | A | 0.67 | rs174566 | G | A | G=A, A=G | 0.95 |
| rs7121958 | G | T | 0.51 | rs60085010 | T | C | T=C, G=T | 0.99 |
| rs10849432 | T | C | 0.90 | rs7964858 | T | C | C=C, T=T | 1.00 |
| rs55990915 | A | C | 0.13 | rs145442994 | CT | C | C=-, A=T | 0.99 |
| rs45597035 | A | G | 0.64 | rs57574208 | AG | A | A=-, G=G | 0.89 |
| rs7993934 | T | C | 0.65 | rs8001158 | G | T | C=T, T=G | 1.00 |
| rs58658771 | A | T | 0.19 | rs2293582 | A | G | T=G, A=A | 0.82 |
| rs9797885 | G | A | 0.71 | rs2317130 | T | C | A=C, G=T | 1.00 |
| rs6091213 | C | T | 0.26 | rs6096091 | A | G | T=G, C=A | 0.96 |
| rs1741640 | C | T | 0.77 | rs477859 | G | A | T=A, C=G | 0.86 |

EAF, effect allele frequency. R^2^ indicates correlation coefficient for linkage disequilibrium.

**Table S3.** Univariate analysis of covariate variables in associations with colorectal cancer risk

| **Factor** | **No. cases** | **Person-years** | **HR (95% CI)** |
| --- | --- | --- | --- |
| **Sex** |  |  |  |
| Women | 1,979 | 2,427,118 | 1.00 (ref.) |
| Men | 2,707 | 2,084,290 | 1.57 (1.49-1.67) |
| **First-degree family history of CRC** |  |  |  |
| No | 3,257 | 3,283,310 | 1.00 (ref.) |
| Yes | 478 | 318,028 | 1.37 (1.25-1.51) |
| **CRC screening** |  |  |  |
| Never | 2,950 | 3,091,503 | 1.00 (ref.) |
| Ever | 1,672 | 1,350,127 | 0.95 (0.89-1.01) |
| **Household income (£/year)** |  |  |  |
| Less than 31,000 | 2,227 | 1,826,760 | 1.00 (ref.) |
| 31,000 to <52,000 | 973 | 1,044,060 | 0.96 (0.89-1.04) |
| More than 52,000 | 770 | 1,024,585 | **0.89 (0.82-0.97)** |
| **Education** |  |  |  |
| College, A and AS levels | 1,782 | 1,914,410 | 1.00 (ref.) |
| O levels and GCSEs | 1,022 | 999,559 | 1.03 (0.96-1.12) |
| CSEs, NVQ, HND, HNC, and others | 811 | 785,002 | 1.04 (0.96-1.13) |
| **Smoking status** |  |  |  |
| Never | 2,178 | 2,489,285 | 1.00 (ref.) |
| Former | 2,037 | 1,559,032 | **1.33 (1.25-1.41)** |
| Current | 457 | 448,097 | **1.26 (1.14-1.39)** |
| **Alcohol consumption** |  |  |  |
| Never or rarely | 778 | 756,375 | 1.00 (ref.) |
| Once a month to twice a week | 1,561 | 1,702,620 | 0.99 (0.90-1.07) |
| 3-4 times/week | 1,097 | 1,099,882 | 1.05 (0.95-1.15) |
| Daily or more | 1,243 | 949,482 | **1.26 (1.16-1.38)** |
| **BMI (kg/m^2^)** |  |  |  |
| <25.0 kg/m^2^ | 1,240 | 1,484,865 | 1.00 (ref.) |
| 25.0 to <30 kg/m^2^ | 2,151 | 1,928,901 | **1.24 (1.16-1.33)** |
| ≥30 kg/m^2^ | 1,278 | 1,084,628 | **1.33 (1.23-1.43)** |
| **Moderate or vigorous activity** |  |  |  |
| Insufficient | 1,769 | 1,662,329 | 1.00 (ref.) |
| Sufficient | 2,017 | 1,996,371 | **0.92 (0.86-0.98)** |
| **Regular use of NSAID** |  |  |  |
| No | 1,857 | 1,782,971 | 1.00 (ref.) |
| Yes | 211 | 195,003 | 0.95 (0.82-1.10) |

HR, hazard ratio; CI, confidence interval; CRC, colorectal cancer; BMI, body mass index; NSAID, nonsteroidal anti-inflammatory drug. Bold font indicates significant difference. Bold font indicates significant difference.

**Table S4.** Sex-specific associations of food intakes and colorectal cancer risk

| **Dietary factor** | **Men** | | | **Women** | | |
| --- | --- | --- | --- | --- | --- | --- |
|  | **No. cases** | **Crude HR (95% CI)** | **Adjusted HR (95% CI)** | **No. cases** | **Crude HR (95% CI)** | **Adjusted HR (95% CI)** |
| **WCRF dietary score** |  |  |  |  |  |  |
| 2-3 | 985 | 1.00 (ref.) | 1.00 (ref.) | 1,313 | 1.00 (ref.) | 1.00 (ref.) |
| 0-1 | 1,686 | **1.21 (1.12-1.31)** | **1.18 (1.09-1.28)** | 649 | 1.03 (0.94-1.13) | 1.02 (0.93-1.12) |
| **Red meat (times/week)** |  |  |  |  |  |  |
| <2 | 1,097 | 1.00 (ref.) | 1.00 (ref.) | 989 | 1.00 (ref.) | 1.00 (ref.) |
| 2 to <3 | 805 | 1.06 (0.97-1.16) | 1.03 (0.94-1.13) | 565 | 1.03 (0.93-1.15) | 1.04 (0.94-1.16) |
| ≥3 | 801 | **1.26 (1.15-1.38)** | **1.20 (1.10-1.32)** | 424 | 1.10 (0.98-1.23) | 1.10 (0.98-1.23) |
| P-trend |  | **<0.001** | **<0.001** |  | 0.12 | 0.12 |
| **Processed meat (times/week)** |  |  |  |  |  |  |
| <1 | 604 | 1.00 (ref.) | 1.00 (ref.) | 963 | 1.00 (ref.) | 1.00 (ref.) |
| 1 | 827 | **1.13 (1.02-1.26)** | 1.10 (0.99-1.23) | 596 | 1.04 (0.94-1.15) | 1.04 (0.93-1.15) |
| ≥2 | 1,272 | **1.26 (1.14-1.39)** | **1.20 (1.09-1.32)** | 418 | 1.04 (0.93-1.17) | 1.03 (0.91-1.15) |
| P-trend |  | **0.02** | 0.07 |  | 0.45 | 0.45 |
| **Poultry (times/week)** |  |  |  |  |  |  |
| <1 | 372 | 1.00 (ref.) | 1.00 (ref.) | 331 | 1.00 (ref.) | 1.00 (ref.) |
| 1 | 1,097 | **1.14 (1.02-1.29)** | **1.13 (1.01-1.28)** | 736 | 0.94 (0.83-1.07) | 0.95 (0.83-1.08) |
| ≥2 | 1,234 | **1.13 (1.01-1.27)** | 1.12 (0.99-1.26) | 908 | 0.91 (0.80-1.03) | 0.91 (0.80-1.04) |
| P-trend |  | 0.30 | 0.43 |  | 0.18 | 0.19 |
| **Total fish (times/week)** |  |  |  |  |  |  |
| ≤1 | 642 | 1.00 (ref.) | 1.00 (ref.) | 462 | 1.00 (ref.) | 1.00 (ref.) |
| >1 to ≤2 | 1,327 | 1.08 (0.98-1.19) | 1.09 (0.99-1.20) | 945 | 0.97 (0.87-1.09) | 1.00 (0.89-1.12) |
| >2 | 733 | 0.99 (0.89-1.10) | 1.00 (0.90-1.11) | 570 | 0.90 (0.80-1.02) | 0.92 (0.81-1.04) |
| P-trend |  | 0.42 | 0.50 |  | 0.08 | 0.12 |
| **Milk (100 mL/day)** |  |  |  |  |  |  |
| <2 | 953 | 1.00 (ref.) | 1.00 (ref.) | 687 | 1.00 (ref.) | 1.00 (ref.) |
| 2 to <3 | 1,080 | **0.88 (0.80-0.96)** | **0.90 (0.83-0.99)** | 781 | 0.91 (0.82-1.01) | 0.92 (0.83-1.02) |
| ≥3 | 591 | **0.82 (0.74-0.90)** | **0.85 (0.77-0.94)** | 398 | **0.87 (0.77-0.98)** | **0.87 (0.76-0.98)** |
| P-trend |  | **<0.001** | **0.002** |  | **0.02** | **0.02** |
| **Cheese (times/week)** |  |  |  |  |  |  |
| <2 | 1,019 | 1.00 (ref.) | 1.00 (ref.) | 830 | 1.00 (ref.) | 1.00 (ref.) |
| 2 to 4 | 1,254 | 0.94 (0.86-1.02) | 0.93 (0.86-1.01) | 857 | 1.05 (0.95-1.16) | 1.07 (0.97-1.18) |
| ≥5 | 374 | 1.00 (0.89-1.13) | 1.00 (0.89-1.13) | 235 | 1.07 (0.93-1.24) | 1.11 (0.96-1.28) |
| P-trend |  | 0.67 | 0.68 |  | 0.25 | 0.10 |
| **Total fruit (servings/day)** |  |  |  |  |  |  |
| <2 | 1,154 | 1.00 (ref.) | 1.00 (ref.) | 490 | 1.00 (ref.) | 1.00 (ref.) |
| 2 to <4 | 1,060 | **0.84 (0.78-0.92)** | **0.87 (0.80-0.94)** | 978 | 1.03 (0.92-1.15) | 1.06 (0.95-1.18) |
| ≥4 | 489 | **0.89 (0.80-0.99)** | 0.93 (0.84-1.04) | 509 | 0.99 (0.87-1.12) | 1.02 (0.89-1.15) |
| P-trend |  | **0.02** | 0.13 |  | 0.70 | 0.99 |
| **Total vegetables (servings/day)** |  |  |  |  |  |  |
| <4 | 1,041 | 1.00 (ref.) | 1.00 (ref.) | 608 | 1.00 (ref.) | 1.00 (ref.) |
| <6 | 906 | 1.01 (0.92-1.10) | 1.01 (0.93-1.11) | 708 | 0.97 (0.87-1.08) | 0.98 (0.88-1.09) |
| ≥6 | 730 | 0.97 (0.88-1.07) | 0.97 (0.88-1.06) | 654 | 0.97 (0.87-1.08) | 0.97 (0.87-1.08) |
| P-trend |  | 0.50 | 0.45 |  | 0.72 | 0.64 |
| **Coffee (cups/day)** |  |  |  |  |  |  |
| <1 | 693 | 1.00 (ref.) | 1.00 (ref.) | 565 | 1.00 (ref.) | 1.00 (ref.) |
| 1 to ≤2 | 997 | 0.93 (0.85-1.03) | 0.93 (0.84-1.02) | 808 | 0.99 (0.89-1.10) | 1.01 (0.90-1.12) |
| >2 | 1,010 | 1.05 (0.96-1.16) | 1.03 (0.93-1.14) | 604 | 0.98 (0.88-1.10) | 0.98 (0.87-1.10) |
| P-trend |  | 0.19 | 0.40 |  | 0.78 | 0.64 |
| **Tea (cups/day)** |  |  |  |  |  |  |
| <3 | 1,103 | 1.00 (ref.) | 1.00 (ref.) | 795 | 1.00 (ref.) | 1.00 (ref.) |
| <5 | 803 | 0.93 (0.85-1.02) | 0.95 (0.87-1.04) | 610 | 0.95 (0.85-1.05) | 0.96 (0.87-1.07) |
| ≥5 | 795 | **0.86 (0.79-0.94)** | **0.88 (0.80-0.96)** | 572 | **0.87 (0.79-0.97)** | **0.88 (0.79-0.98)** |
| P-trend |  | **0.001** | **0.01** |  | **0.01** | **0.02** |
| **Alcohol (times/week)** |  |  |  |  |  |  |
| <1 | 464 | 1.00 (ref.) | 1.00 (ref.) | 748 | 1.00 (ref.) | 1.00 (ref.) |
| 1 to 2 | 635 | 1.06 (0.94-1.19) | 1.07 (0.95-1.20) | 492 | 0.91 (0.81-1.02) | 0.92 (0.82-1.03) |
| ≥3 | 1,607 | **1.22 (1.10-1.35)** | **1.25 (1.12-1.39)** | 733 | 0.91 (0.82-1.01) | 0.92 (0.83-1.02) |
| P-trend |  | **<0.001** | **<0.001** |  | 0.09 | 0.15 |
| **IV-weighted dietary score** |  |  |  |  |  |  |
| -13.22 to <-2.88 | 550 | 1.00 (ref.) | 1.00 (ref.) | 698 | 1.00 (ref.) | 1.00 (ref.) |
| -2.88 to <1.09 | 832 | **1.27 (1.14-1.41)** | **1.26 (1.13-1.40)** | 617 | 1.04 (0.93-1.15) | 1.04 (0.93-1.15) |
| 1.09 to 13.02 | 1,155 | **1.45 (1.31-1.60)** | **1.42 (1.28-1.57)** | 501 | 1.11 (0.99-1.24) | 1.11 (0.99-1.24) |
| P-trend |  | **<0.001** | **<0.001** |  | 0.085 | 0.09 |

The HRs were estimated using Cox proportional hazard models with adjustment for first-degree family history of colorectal cancer, smoking status, alcohol consumption (except when alcohol intake and dietary score are exposure), body mass index, and physical activity. HR, hazard ratio; CI, confidence interval; WCRF, World Cancer Research Fund; IV, inverse variance. Bold font indicates significant difference.

**Table S5.** Site-specific associations of food intakes and colorectal cancer risk

| **Dietary factor** | **Colon cancer** | | | **Rectal cancer** | | |
| --- | --- | --- | --- | --- | --- | --- |
|  | **No. cases** | **Crude HR (95% CI)** | **Adjusted HR (95% CI)** | **No. cases** | **Crude HR (95% CI)** | **Adjusted HR (95% CI)** |
| **WCRF dietary score** |  |  |  |  |  |  |
| 2-3 | 1,586 | 1.00 (ref.) | 1.00 (ref.) | 712 | 1.00 (ref.) | 1.00 (ref.) |
| 0-1 | 1,511 | **1.19 (1.11-1.28)** | **1.09 (1.01-1.17)** | 824 | **1.43 (1.30-1.58)** | **1.18 (1.07-1.31)** |
| **Red meat (times/week)** |  |  |  |  |  |  |
| <2 | 1,400 | 1.00 (ref.) | 1.00 (ref.) | 686 | 1.00 (ref.) | 1.00 (ref.) |
| 2 to <3 | 908 | 1.05 (0.97-1.14) | 1.03 (0.95-1.12) | 462 | 1.10 (0.98-1.24) | 1.04 (0.93-1.18) |
| ≥3 | 819 | **1.23 (1.13-1.34)** | **1.17 (1.07-1.28)** | 406 | **1.27 (1.12-1.43)** | **1.14 (1.01-1.30)** |
| P-trend |  | **<0.001** | **<0.001** |  | **<0.001** | **0.03** |
| **Processed meat (times/week)** |  |  |  |  |  |  |
| <1 | 1,077 | 1.00 (ref.) | 1.00 (ref.) | 490 | 1.00 (ref.) | 1.00 (ref.) |
| 1 | 961 | **1.14 (1.04-1.24)** | 1.07 (0.98-1.17) | 462 | **1.20 (1.06-1.37)** | 1.05 (0.92-1.19) |
| ≥2 | 1,089 | **1.25 (1.15-1.36)** | **1.11 (1.01-1.21)** | 601 | **1.51 (1.34-1.71)** | **1.16 (1.03-1.32)** |
| P-trend |  | **0.004** | 0.08 |  | **0.004** | 0.45 |
| **Poultry (times/week)** |  |  |  |  |  |  |
| <1 | 485 | 1.00 (ref.) | 1.00 (ref.) | 218 | 1.00 (ref.) | 1.00 (ref.) |
| 1 | 1,225 | 1.02 (0.92-1.14) | 1.02 (0.92-1.13) | 608 | 1.14 (0.97-1.33) | 1.11 (0.95-1.30) |
| ≥2 | 1,417 | 0.99 (0.89-1.09) | 0.98 (0.89-1.09) | 725 | 1.11 (0.95-1.29) | 1.10 (0.94-1.28) |
| P-trend |  | 0.45 | 0.46 |  | 0.69 | 0.60 |
| **Total fish (times/week)** |  |  |  |  |  |  |
| ≤1 | 749 | 1.00 (ref.) | 1.00 (ref.) | 355 | 1.00 (ref.) | 1.00 (ref.) |
| >1 to ≤2 | 1,519 | 1.00 (0.91-1.09) | 1.03 (0.94-1.13) | 753 | 1.07 (0.95-1.22) | 1.10 (0.96-1.24) |
| >2 | 859 | **0.89 (0.81-0.98)** | 0.93 (0.84-1.02) | 444 | 1.01 (0.88-1.17) | 1.04 (0.90-1.20) |
| P-trend |  | **0.01** | 0.05 |  | 0.84 | 0.91 |
| **Milk (100 mL/day)** |  |  |  |  |  |  |
| <2 | 1,095 | 1.00 (ref.) | 1.00 (ref.) | 545 | 1.00 (ref.) | 1.00 (ref.) |
| 2 to <3 | 1,257 | **0.90 (0.83-0.97)** | **0.92 (0.84-0.99)** | 604 | **0.88 (0.78-0.99)** | 0.89 (0.79-1.00) |
| ≥3 | 652 | **0.83 (0.75-0.92)** | **0.84 (0.76-0.93)** | 337 | 0.88 (0.77-1.00) | 0.88 (0.77-1.01) |
| P-trend |  | **<0.001** | **<0.001** |  | **0.04** | 0.05 |
| **Cheese (times/week)** |  |  |  |  |  |  |
| <2 | 1,253 | 1.00 (ref.) | 1.00 (ref.) | 596 | 1.00 (ref.) | 1.00 (ref.) |
| 2 to 4 | 1,415 | 1.01 (0.94-1.09) | 1.00 (0.93-1.08) | 696 | 1.04 (0.93-1.16) | 0.98 (0.87-1.09) |
| ≥5 | 391 | 1.02 (0.91-1.15) | 1.02 (0.91-1.14) | 218 | **1.18 (1.01-1.38)** | 1.11 (0.95-1.30) |
| P-trend |  | 0.68 | 0.82 |  | 0.05 | 0.34 |
| **Total fruit (servings/day)** |  |  |  |  |  |  |
| <2 | 1,051 | 1.00 (ref.) | 1.00 (ref.) | 593 | 1.00 (ref.) | 1.00 (ref.) |
| 2 to <4 | 1,381 | **0.90 (0.83-0.98)** | 0.97 (0.90-1.05) | 657 | **0.77 (0.69-0.86)** | **0.87 (0.77-0.97)** |
| ≥4 | 694 | **0.90 (0.82-0.99)** | 1.00 (0.91-1.11) | 304 | **0.72 (0.62-0.82)** | **0.85 (0.74-0.98)** |
| P-trend |  | **0.04** | 0.90 |  | **<0.001** | **0.03** |
| **Total vegetables (servings/day)** |  |  |  |  |  |  |
| <4 | 1,120 | 1.00 (ref.) | 1.00 (ref.) | 529 | 1.00 (ref.) | 1.00 (ref.) |
| 4 to <6 | 1,073 | 0.93 (0.85-1.01) | 0.97 (0.89-1.05) | 541 | 1.01 (0.89-1.14) | 1.06 (0.94-1.20) |
| ≥6 | 916 | **0.90 (0.82-0.98)** | 0.93 (0.85-1.02) | 468 | 0.99 (0.87-1.12) | 1.05 (0.92-1.19) |
| P-trend |  | **0.02** | 0.13 |  | 0.81 | 0.54 |
| **Coffee (cups/day)** |  |  |  |  |  |  |
| <1 | 845 | 1.00 (ref.) | 1.00 (ref.) | 413 | 1.00 (ref.) | 1.00 (ref.) |
| 1 to ≤2 | 1,190 | 0.94 (0.86-1.02) | 0.94 (0.86-1.03) | 615 | 1.01 (0.89-1.14) | 0.99 (0.87-1.12) |
| >2 | 1,091 | 1.05 (0.96-1.15) | 1.02 (0.94-1.12) | 523 | 1.04 (0.91-1.18) | 0.97 (0.85-1.11) |
| P-trend |  | 0.05 | 0.24 |  | 0.52 | 0.67 |
| **Tea (cups/day)** |  |  |  |  |  |  |
| <3 | 1,275 | 1.00 (ref.) | 1.00 (ref.) | 623 | 1.00 (ref.) | 1.00 (ref.) |
| 3 to <5 | 942 | 0.92 (0.85-1.00) | 0.95 (0.87-1.03) | 471 | 0.96 (0.85-1.08) | 0.97 (0.86-1.10) |
| ≥5 | 908 | **0.86 (0.79-0.93)** | **0.86 (0.79-0.94)** | 459 | 0.89 (0.79-1.01) | 0.90 (0.80-1.02) |
| P-trend |  | **<0.001** | **0.001** |  | 0.064 | 0.09 |
| **Alcohol (times/week)** |  |  |  |  |  |  |
| <1 | 870 | 1.00 (ref.) | 1.00 (ref.) | 342 | 1.00 (ref.) | 1.00 (ref.) |
| 1 to 2 | 743 | 0.95 (0.86-1.05) | 0.92 (0.84-1.02) | 384 | **1.23 (1.06-1.42)** | 1.12 (0.97-1.30) |
| ≥3 | 1,512 | 1.06 (0.98-1.15) | 1.01 (0.93-1.11) | 828 | **1.47 (1.30-1.67)** | **1.26 (1.11-1.44)** |
| P-trend |  | 0.06 | 0.45 |  | **<0.001** | **<0.001** |
| **IV-weighted dietary score** |  |  |  |  |  |  |
| -13.22 to <-2.88 | 850 | 1.00 (ref.) | 1.00 (ref.) | 398 | 1.00 (ref.) | 1.00 (ref.) |
| -2.88 to <1.09 | 996 | **1.20 (1.09-1.31)** | **1.16 (1.06-1.28)** | 453 | **1.16 (1.01-1.33)** | 1.09 (0.95-1.25) |
| 1.09 to 13.02 | 1,076 | **1.34 (1.22-1.46)** | **1.25 (1.14-1.37)** | 580 | **1.52 (1.34-1.73)** | **1.31 (1.15-1.50)** |
| P-trend |  | **<0.001** | **<0.001** |  | **<0.001** | **<0.001** |

The HRs were estimated using Cox proportional hazard models with adjustment for sex, first-degree family history of colorectal cancer, smoking status, alcohol consumption (except when alcohol intake and dietary score are exposure), body mass index, and physical activity. HR, hazard ratio; CI, confidence interval; WCRF, World Cancer Research Fund; IV, inverse variance. Bold font indicates significant difference.

**Table S6.** Associations of each polygenic risk score with colorectal cancer risk

| **Decile** | **Unweighted PRS** | | **Standard weighted PRS** | | **Inverse-variance weighted PRS** | |
| --- | --- | --- | --- | --- | --- | --- |
|  | **No. cases** | **HR (95% CI)** | **No. cases** | **HR (95% CI)** | **No. cases** | **HR (95% CI)** |
| **Total (N=374,004)** | | | | | | |
| Tertile 1 | 1,119 | 1.00 (ref.) | 1,054 | 1.00 (ref.) | 1,039 | 1.00 (ref.) |
| Tertile 2 | 1,464 | 1.40 (1.29-1.51) | 1,452 | 1.38 (1.27-1.49) | 1,455 | 1.40 (1.30-1.52) |
| Tertile 3 | 2,103 | 1.98 (1.84-2.13) | 2,180 | 2.09 (1.94-2.24) | 2,192 | 2.12 (1.97-2.29) |
| Decile 1 | 286 | 1.00 (ref.) | 229 | 1.00 (ref.) | 215 | 1.00 (ref.) |
| Decile 2 | 415 | 1.28 (1.10-1.49) | 325 | 1.43 (1.21-1.69) | 345 | 1.60 (1.35-1.90) |
| Decile 3 | 418 | 1.40 (1.20-1.62) | 364 | 1.58 (1.34-1.87) | 346 | 1.60 (1.35-1.90) |
| Decile 4 | 254 | 1.52 (1.28-1.80) | 389 | 1.71 (1.45-2.02) | 408 | 1.90 (1.61-2.24) |
| Decile 5 | 602 | 1.73 (1.50-1.99) | 424 | 1.87 (1.59-2.20) | 403 | 1.88 (1.59-2.22) |
| Decile 6 | 300 | 1.75 (1.49-2.06) | 449 | 1.96 (1.67-2.30) | 460 | 2.15 (1.82-2.52) |
| Decile 7 | 596 | 1.88 (1.63-2.16) | 497 | 2.18 (1.87-2.55) | 497 | 2.31 (1.97-2.71) |
| Decile 8 | 554 | 2.16 (1.87-2.49) | 536 | 2.36 (2.03-2.76) | 552 | 2.57 (2.20-3.01) |
| Decile 9 | 635 | 2.45 (2.13-2.81) | 648 | 2.86 (2.46-3.32) | 634 | 2.97 (2.54-3.46) |
| Decile 10 | 626 | 3.23 (2.81-3.71) | 825 | 3.66 (3.16-4.24) | 826 | 3.87 (3.33-4.50) |
| **Men (N=174,576)** | | | | | | |
| Tertile 1 | 630 | 1.00 (ref.) | 588 | 1.00 (ref.) | 571 | 1.00 (ref.) |
| Tertile 2 | 843 | 1.42 (1.28-1.58) | 854 | 1.46 (1.31-1.62) | 843 | 1.49 (1.34-1.66) |
| Tertile 3 | 1,234 | 2.07 (1.88-2.28) | 1,265 | 2.19 (1.99-2.41) | 1,293 | 2.30 (2.09-2.54) |
| Decile 1 | 161 | 1.00 (ref.) | 128 | 1.00 (ref.) | 113 | 1.00 (ref.) |
| Decile 2 | 231 | 1.28 (1.04-1.56) | 166 | 1.31 (1.04-1.65) | 184 | 1.63 (1.29-2.06) |
| Decile 3 | 238 | 1.44 (1.18-1.76) | 207 | 1.63 (1.31-2.03) | 201 | 1.78 (1.42-2.25) |
| Decile 4 | 147 | 1.57 (1.25-1.96) | 238 | 1.91 (1.54-2.37) | 235 | 2.10 (1.68-2.63) |
| Decile 5 | 344 | 1.76 (1.46-2.12) | 241 | 1.93 (1.55-2.39) | 241 | 2.17 (1.74-2.72) |
| Decile 6 | 164 | 1.73 (1.39-2.15) | 265 | 2.08 (1.69-2.57) | 264 | 2.35 (1.89-2.93) |
| Decile 7 | 364 | 2.04 (1.69-2.45) | 301 | 2.37 (1.92-2.91) | 284 | 2.53 (2.03-3.14) |
| Decile 8 | 354 | 2.48 (2.06-2.98) | 313 | 2.52 (2.05-3.09) | 356 | 3.19 (2.58-3.95) |
| Decile 9 | 361 | 2.51 (2.08-3.02) | 384 | 3.07 (2.51-3.75) | 360 | 3.25 (2.63-4.01) |
| Decile 10 | 343 | 3.21 (2.66-3.88) | 464 | 3.76 (3.09-4.58) | 469 | 4.26 (3.47-5.23) |
| **Women (N=199,428)** | | | | | | |
| Tertile 1 | 489 | 1.00 (ref.) | 466 | 1.00 (ref.) | 468 | 1.00 (ref.) |
| Tertile 2 | 621 | 1.36 (1.21-1.53) | 598 | 1.28 (1.13-1.44) | 612 | 1.30 (1.15-1.47) |
| Tertile 3 | 869 | 1.86 (1.67-2.08) | 915 | 1.95 (1.75-2.19) | 899 | 1.91 (1.70-2.13) |
| Decile 1 | 125 | 1.00 (ref.) | 101 | 1.00 (ref.) | 102 | 1.00 (ref.) |
| Decile 2 | 184 | 1.28 (1.02-1.61) | 159 | 1.57 (1.22-2.02) | 161 | 1.57 (1.22-2.01) |
| Decile 3 | 180 | 1.34 (1.07-1.69) | 157 | 1.53 (1.19-1.96) | 145 | 1.40 (1.09-1.81) |
| Decile 4 | 107 | 1.45 (1.12-1.88) | 151 | 1.47 (1.14-1.89) | 173 | 1.68 (1.31-2.15) |
| Decile 5 | 258 | 1.69 (1.36-2.09) | 183 | 1.80 (1.41-2.29) | 162 | 1.56 (1.22-2.00) |
| Decile 6 | 136 | 1.77 (1.39-2.25) | 184 | 1.80 (1.41-2.29) | 196 | 1.92 (1.51-2.43) |
| Decile 7 | 232 | 1.67 (1.34-2.07) | 196 | 1.95 (1.53-2.47) | 213 | 2.07 (1.64-2.63) |
| Decile 8 | 200 | 1.76 (1.41-2.20) | 223 | 2.17 (1.72-2.75) | 196 | 1.89 (1.49-2.41) |
| Decile 9 | 274 | 2.37 (1.92-2.93) | 264 | 2.59 (2.06-3.26) | 274 | 2.66 (2.12-3.33) |
| Decile 10 | 283 | 3.24 (2.62-4.00) | 361 | 3.54 (2.84-4.41) | 357 | 3.45 (2.77-4.30) |
| **Colon cancer (N=374,004)** | | | | | | |
| Tertile 1 | 754 | 1.00 (ref.) | 711 | 1.00 (ref.) | 709 | 1.00 (ref.) |
| Tertile 2 | 952 | 1.35 (1.23-1.48) | 975 | 1.37 (1.25-1.51) | 960 | 1.36 (1.23-1.49) |
| Tertile 3 | 1,425 | 1.99 (1.82-2.17) | 1,445 | 2.04 (1.87-2.24) | 1,462 | 2.07 (1.89-2.26) |
| Decile 1 | 186 | 1.00 (ref.) | 157 | 1.00 (ref.) | 153 | 1.00 (ref.) |
| Decile 2 | 276 | 1.31 (1.09-1.57) | 230 | 1.47 (1.20-1.81) | 248 | 1.62 (1.32-1.98) |
| Decile 3 | 292 | 1.50 (1.24-1.80) | 238 | 1.51 (1.23-1.84) | 217 | 1.41 (1.15-1.74) |
| Decile 4 | 168 | 1.54 (1.25-1.90) | 258 | 1.65 (1.35-2.01) | 272 | 1.78 (1.46-2.17) |
| Decile 5 | 389 | 1.71 (1.44-2.04) | 285 | 1.83 (1.50-2.22) | 279 | 1.82 (1.50-2.22) |
| Decile 6 | 185 | 1.65 (1.35-2.03) | 296 | 1.88 (1.55-2.28) | 294 | 1.92 (1.58-2.34) |
| Decile 7 | 413 | 1.99 (1.68-2.37) | 330 | 2.11 (1.74-2.55) | 323 | 2.11 (1.74-2.55) |
| Decile 8 | 361 | 2.16 (1.81-2.58) | 359 | 2.30 (1.91-2.78) | 367 | 2.40 (1.98-2.89) |
| Decile 9 | 439 | 2.59 (2.18-3.08) | 416 | 2.67 (2.22-3.20) | 428 | 2.81 (2.33-3.38) |
| Decile 10 | 422 | 3.33 (2.81-3.96) | 562 | 3.62 (3.04-4.33) | 550 | 3.61 (3.02-4.32) |
| **Rectal cancer (N=374,004)** | | | | | | |
| Tertile 1 | 365 | 1.00 (ref.) | 343 | 1.00 (ref.) | 330 | 1.00 (ref.) |
| Tertile 2 | 512 | 1.50 (1.31-1.71) | 477 | 1.40 (1.22-1.60) | 495 | 1.51 (1.31-1.73) |
| Tertile 3 | 678 | 1.96 (1.73-2.73) | 735 | 2.17 (1.91-2.47) | 730 | 2.24 (1.97-2.55) |
| Decile 1 | 100 | 1.00 (ref.) | 72 | 1.00 (ref.) | 62 | 1.00 (ref.) |
| Decile 2 | 139 | 1.23 (0.95-1.59) | 95 | 1.33 (0.98-1.80) | 97 | 1.57 (1.14-2.15) |
| Decile 3 | 126 | 1.21 (0.93-1.58) | 126 | 1.75 (1.31-2.34) | 129 | 2.08 (1.54-2.82) |
| Decile 4 | 86 | 1.47 (1.10-1.97) | 131 | 1.84 (1.38-2.46) | 136 | 2.21 (1.63-2.98) |
| Decile 5 | 213 | 1.75 (1.38-2.22) | 139 | 1.96 (1.47-2.60) | 124 | 2.02 (1.49-2.73) |
| Decile 6 | 115 | 1.93 (1.48-2.52) | 153 | 2.13 (1.61-2.82) | 166 | 2.69 (2.01-3.60) |
| Decile 7 | 183 | 1.65 (1.30-2.11) | 167 | 2.34 (1.78-3.09) | 174 | 2.82 (2.11-3.77) |
| Decile 8 | 193 | 2.17 (1.70-2.76) | 177 | 2.50 (1.90-3.29) | 185 | 3.01 (2.26-4.01) |
| Decile 9 | 196 | 2.18 (1.71-2.77) | 232 | 3.27 (2.51-4.26) | 296 | 3.37 (2.53-4.47) |
| Decile 10 | 204 | 3.03 (2.39-3.85) | 263 | 3.75 (2.89-4.86) | 276 | 4.53 (3.44-5.97) |

Multivariable regression models are adjusted for sex (except for sex-specific analyses) and first-degree family history of colorectal cancer.

**Table S7.** Joint effects of dietary intake and polygenic risk score on colorectal cancer risk in men and women

| **Dietary factor** | **Low PRS** | | | **Intermediate PRS** | | | **High PRS** | | | **P_interaction_** |
| --- | --- | --- | --- | --- | --- | --- | --- | --- | --- | --- |
|  | **No. cases** | **Person-years** | **HR (95% CI)** | **No. cases** | **Person-years** | **HR (95% CI)** | **No. cases** | **Person-years** | **HR (95% CI)** |  |
| **Men** | | | | | | | | | | |
| **WCRF dietary score** |  |  |  |  |  |  |  |  |  |  |
| 2-3 | 212 | 280,654 | 1.00 (ref.) | 298 | 278,517 | 1.41 (1.18-1.68) | 475 | 272,945 | 2.30 (1.95-2.70) | 0.58 |
| 0-1 | 351 | 413,030 | 1.15 (0.97-1.36) | 530 | 407,517 | 1.75 (1.49-2.05) | 805 | 409,256 | 2.66 (2.29-3.10) |  |
| **Red meat (times/week)** |  |  |  |  |  |  |  |  |  |  |
| <2 | 236 | 319,398 | 1.00 (ref.) | 337 | 314,059 | 1.44 (1.22-1.71) | 524 | 309,267 | 2.30 (1.98-2.69) | 0.91 |
| 2 to <3 | 165 | 210,927 | 0.99 (0.81-1.21) | 248 | 206,697 | 1.51 (1.27-1.81) | 392 | 208,156 | 2.37 (2.02-2.79) |  |
| ≥3 | 169 | 170,172 | 1.20 (0.99-1.46) | 257 | 171,349 | 1.82 (1.53-2.18) | 375 | 171,166 | 2.65 (2.25-3.12) |  |
| **Processed meat (times/week)** |  |  |  |  |  |  |  |  |  |  |
| <1 | 124 | 175,434 | 1.00 (ref.) | 188 | 176,381 | 1.50 (1.20-1.88) | 292 | 170,152 | 2.42 (1.96-2.99) | 0.96 |
| 1 | 175 | 212,780 | 1.14 (0.91-1.44) | 254 | 210,270 | 1.67 (1.35-2.07) | 398 | 212,444 | 2.60 (2.13-3.19) |  |
| ≥2 | 270 | 312,194 | 1.23 (1.00-1.52) | 401 | 305,604 | 1.87 (1.53-2.28) | 601 | 306,044 | 2.79 (2.30-3.39) |  |
| **Poultry (times/week)** |  |  |  |  |  |  |  |  |  |  |
| ≥2 | 248 | 331,696 | 1.00 (ref.) | 382 | 329,205 | 1.54 (1.31-1.81) | 604 | 327,992 | 2.44 (2.11-2.83) | 0.81 |
| 1 | 243 | 264,619 | 1.11 (0.93-1.32) | 339 | 261,195 | 1.56 (1.33-1.84) | 515 | 259,261 | 2.40 (2.06-2.79) |  |
| <1 | 80 | 103,953 | 0.94 (0.73-1.21) | 120 | 101,618 | 1.45 (1.16-1.80) | 172 | 100,952 | 2.10 (1.73-2.55) |  |
| **Total fish (times/week)** |  |  |  |  |  |  |  |  |  |  |
| >2 | 157 | 190,310 | 1.00 (ref.) | 225 | 184,691 | 1.46 (1.19-1.79) | 351 | 185,137 | 2.29 (1.90-2.77) | 0.83 |
| >1 to ≤2 | 274 | 319,633 | 1.06 (0.87-1.29) | 426 | 317,963 | 1.66 (1.38-2.00) | 627 | 316,783 | 2.46 (2.06-2.92) |  |
| ≤1 | 139 | 190,490 | 1.03 (0.82-1.29) | 191 | 189,446 | 1.41 (1.14-1.74) | 312 | 186,869 | 2.33 (1.93-2.83) |  |
| **Milk (100 mL/day)** |  |  |  |  |  |  |  |  |  |  |
| ≥3 | 116 | 164,968 | 1.00 (ref.) | 188 | 162,140 | 1.63 (1.29-2.05) | 287 | 161,308 | 2.50 (2.01-3.10) | 0.64 |
| 2 to <3 | 234 | 279,068 | 1.16 (0.93-1.45) | 345 | 274,903 | 1.75 (1.42-2.16) | 501 | 275,108 | 2.54 (2.08-3.11) |  |
| <2 | 206 | 231,356 | 1.29 (1.03-1.63) | 284 | 229,786 | 1.79 (1.44-2.22) | 463 | 227,658 | 2.95 (2.41-3.62) |  |
| **Cheese (times/week)** |  |  |  |  |  |  |  |  |  |  |
| <2 | 207 | 252,822 | 1.00 (ref.) | 304 | 248,438 | 1.48 (1.24-1.77) | 508 | 247,035 | 2.50 (2.13-2.94) | 0.31 |
| 2 to 4 | 279 | 337,864 | 1.02 (0.85-1.22) | 404 | 333,130 | 1.50 (1.27-1.77) | 571 | 333,022 | 2.12 (1.81-2.49) |  |
| ≥5 | 79 | 96,122 | 1.05 (0.81-1.36) | 113 | 96,529 | 1.50 (1.19-1.88) | 182 | 94,564 | 2.46 (2.01-3.00) |  |
| **Total fruit (servings/day)** |  |  |  |  |  |  |  |  |  |  |
| ≥4 | 121 | 124,752 | 1.00 (ref.) | 154 | 120,754 | 1.31 (1.03-1.66) | 214 | 120,527 | 1.83 (1.46-2.28) | 0.18 |
| 2 to <4 | 215 | 292,017 | 0.77 (0.62-0.97) | 321 | 290,692 | 1.15 (0.93-1.42) | 524 | 285,913 | 1.92 (1.58-2.34) |  |
| <2 | 234 | 283,312 | 0.89 (0.71-1.11) | 366 | 280,323 | 1.41 (1.15-1.73) | 554 | 281,700 | 2.12 (1.74-2.58) |  |
| **Total vegetables (servings/day)** |  |  |  |  |  |  |  |  |  |  |
| ≥6 | 162 | 185,545 | 1.00 (ref.) | 199 | 182,472 | 1.25 (1.02-1.54) | 369 | 180,143 | 2.34 (1.95-2.82) | 0.16 |
| <6 | 191 | 226,352 | 1.00 (0.81-1.24) | 294 | 224,139 | 1.55 (1.28-1.87) | 421 | 221,632 | 2.25 (1.88-2.70) |  |
| <4 | 213 | 283,822 | 0.97 (0.79-1.19) | 337 | 281,115 | 1.53 (1.27-1.85) | 491 | 282,054 | 2.24 (1.87-2.68) |  |
| **Coffee (cups/day)** |  |  |  |  |  |  |  |  |  |  |
| >2 | 228 | 255,923 | 1.00 (ref.) | 306 | 251,128 | 1.37 (1.15-1.62) | 366 | 217,714 | 2.16 (1.84-2.53) | 0.29 |
| 1 to ≤2 | 188 | 263,594 | 0.76 (0.63-0.92) | 323 | 260,653 | 1.32 (1.11-1.56) | 386 | 197,079 | 1.99 (1.70-2.33) |  |
| <1 | 154 | 180,872 | 0.97 (0.79-1.20) | 213 | 180,222 | 1.34 (1.11-1.62) | 537 | 273,580 | 2.06 (1.74-2.44) |  |
| **Tea (cups/day)** |  |  |  |  |  |  |  |  |  |  |
| ≥5 | 173 | 218,265 | 1.00 (ref.) | 256 | 218,686 | 1.47 (1.21-1.78) | 476 | 247,968 | 2.10 (1.76-2.52) | 0.36 |
| <5 | 159 | 203,484 | 0.95 (0.77-1.18) | 258 | 195,804 | 1.61 (1.33-1.96) | 486 | 259,260 | 2.39 (2.00-2.86) |  |
| <3 | 238 | 278,496 | 1.12 (0.92-1.36) | 328 | 277,447 | 1.54 (1.28-1.85) | 326 | 180,950 | 2.57 (2.17-3.06) |  |
| **Alcohol (times/week)** |  |  |  |  |  |  |  |  |  |  |
| <1 | 92 | 138,552 | 1.00 (ref.) | 147 | 138,236 | 1.61 (1.24-2.09) | 225 | 137,138 | 2.46 (1.93-3.14) | 0.96 |
| 1 to 2 | 133 | 184,552 | 1.14 (0.87-1.48) | 202 | 183,871 | 1.71 (1.34-2.19) | 300 | 184,011 | 2.56 (2.03-3.24) |  |
| ≥3 | 345 | 378,013 | 1.34 (1.06-1.69) | 494 | 370,458 | 1.95 (1.56-2.44) | 768 | 367,834 | 3.06 (2.46-3.80) |  |
| **IV-weighted dietary score** |  |  |  |  |  |  |  |  |  |  |
| -13.22 to <-2.88 | 119 | 176,079 | 1.00 (ref.) | 169 | 174,993 | 1.42 (1.13-1.80) | 262 | 174,139 | 2.19 (1.77-2.73) | 0.75 |
| -2.88 to <1.09 | 172 | 214,223 | 1.19 (0.94-1.50) | 249 | 211,445 | 1.73 (1.39-2.16) | 411 | 208,777 | 2.93 (2.39-3.59) |  |
| 1.09 to 13.02 | 250 | 266,476 | 1.41 (1.13-1.75) | 364 | 263,353 | 2.08 (1.69-2.55) | 541 | 262,981 | 3.09 (2.54-3.77) |  |
| **Women** | | | | | | | | | | |
| **WCRF dietary score** |  |  |  |  |  |  |  |  |  |  |
| 2-3 | 307 | 532,269 | 1.00 (ref.) | 409 | 532,462 | 1.33 (1.15-1.54) | 597 | 533,747 | 1.94 (1.69-2.22) | 0.70 |
| 0-1 | 158 | 268,871 | 1.08 (0.89-1.31) | 199 | 273,639 | 1.33 (1.11-1.59) | 292 | 271,929 | 1.95 (1.66-2.29) |  |
| **Red meat (times/week)** |  |  |  |  |  |  |  |  |  |  |
| <2 | 226 | 428,868 | 1.00 (ref.) | 298 | 425,579 | 1.33 (1.12-1.58) | 465 | 427,887 | 2.06 (1.76-2.42) | 0.46 |
| 2 to <3 | 137 | 222,111 | 1.12 (0.91-1.39) | 189 | 228,246 | 1.50 (1.24-1.82) | 239 | 224,132 | 1.94 (1.62-2.33) |  |
| ≥3 | 105 | 153,855 | 1.21 (0.96-1.53) | 125 | 156,083 | 1.42 (1.14-1.77) | 194 | 157,524 | 2.17 (1.79-2.63) |  |
| **Processed meat (times/week)** |  |  |  |  |  |  |  |  |  |  |
| <1 | 221 | 397,950 | 1.00 (ref.) | 293 | 397,988 | 1.32 (1.11-1.57) | 449 | 398,617 | 2.01 (1.71-2.37) | 0.23 |
| 1 | 144 | 236,047 | 1.10 (0.89-1.35) | 201 | 240,593 | 1.51 (1.24-1.82) | 251 | 240,063 | 1.88 (1.57-2.26) |  |
| ≥2 | 103 | 170,744 | 1.10 (0.87-1.39) | 116 | 171,179 | 1.23 (0.98-1.54) | 199 | 170,773 | 2.13 (1.76-2.58) |  |
| **Poultry (times/week)** |  |  |  |  |  |  |  |  |  |  |
| ≥2 | 215 | 390,013 | 1.00 (ref.) | 294 | 398,925 | 1.34 (1.12-1.60) | 399 | 395,904 | 1.83 (1.55-2.16) | 0.23 |
| 1 | 176 | 287,001 | 1.04 (0.85-1.27) | 230 | 284,286 | 1.37 (1.13-1.65) | 330 | 287,352 | 1.93 (1.63-2.30) |  |
| <1 | 77 | 127,709 | 1.06 (0.82-1.38) | 87 | 126,619 | 1.20 (0.93-1.53) | 167 | 126,233 | 2.32 (1.89-2.84) |  |
| **Total fish (times/week)** |  |  |  |  |  |  |  |  |  |  |
| >2 | 133 | 234,216 | 1.00 (ref.) | 180 | 233,746 | 1.36 (1.08-1.70) | 257 | 231,633 | 1.95 (1.58-2.41) | 0.89 |
| >1 to ≤2 | 221 | 371,141 | 1.11 (0.89-1.37) | 285 | 375,596 | 1.40 (1.14-1.72) | 439 | 376,333 | 2.16 (1.78-2.62) |  |
| ≤1 | 113 | 199,606 | 1.15 (0.90-1.48) | 146 | 200,775 | 1.48 (1.17-1.87) | 203 | 201,768 | 2.04 (1.63-2.54) |  |
| **Milk (100 mL/day)** |  |  |  |  |  |  |  |  |  |  |
| ≥3 | 98 | 165,866 | 1.00 (ref.) | 105 | 166,239 | 1.07 (0.81-1.41) | 195 | 168,192 | 1.95 (1.53-2.49) | 0.21 |
| 2 to <3 | 190 | 317,093 | 1.05 (0.82-1.34) | 252 | 318,176 | 1.37 (1.09-1.74) | 339 | 317,629 | 1.85 (1.48-2.32) |  |
| <2 | 155 | 272,369 | 1.05 (0.82-1.36) | 226 | 275,211 | 1.53 (1.20-1.93) | 306 | 273,655 | 2.08 (1.66-2.62) |  |
| **Cheese (times/week)** |  |  |  |  |  |  |  |  |  |  |
| <2 | 190 | 341,538 | 1.00 (ref.) | 254 | 344,907 | 1.32 (1.10-1.60) | 386 | 345,797 | 2.01 (1.69-2.39) | 0.70 |
| 2 to 4 | 214 | 348,079 | 1.17 (0.96-1.43) | 266 | 349,662 | 1.44 (1.20-1.74) | 377 | 349,962 | 2.04 (1.71-2.43) |  |
| ≥5 | 53 | 97,351 | 1.08 (0.79-1.46) | 70 | 97,282 | 1.42 (1.08-1.87) | 112 | 96,015 | 2.31 (1.83-2.92) |  |
| **Total fruit (servings/day)** |  |  |  |  |  |  |  |  |  |  |
| ≥4 | 113 | 196,804 | 1.00 (ref.) | 154 | 197,155 | 1.35 (1.06-1.72) | 242 | 197,673 | 2.12 (1.69-2.65) | 0.56 |
| 2 to <4 | 235 | 390,767 | 1.12 (0.90-1.40) | 296 | 390,341 | 1.42 (1.14-1.76) | 447 | 390,481 | 2.14 (1.74-2.63) |  |
| <2 | 120 | 217,501 | 1.10 (0.85-1.42) | 162 | 222,598 | 1.44 (1.13-1.84) | 208 | 221,606 | 1.86 (1.48-2.34) |  |
| **Total vegetables (servings/day)** |  |  |  |  |  |  |  |  |  |  |
| ≥6 | 170 | 264,878 | 1.00 (ref.) | 187 | 263,468 | 1.10 (0.90-1.36) | 297 | 262,462 | 1.77 (1.46-2.14) | 0.37 |
| <6 | 158 | 288,836 | 0.88 (0.71-1.09) | 222 | 288,150 | 1.23 (1.01-1.50) | 328 | 292,069 | 1.79 (1.48-2.15) |  |
| <4 | 137 | 249,360 | 0.91 (0.73-1.14) | 203 | 256,729 | 1.31 (1.07-1.61) | 268 | 252,951 | 1.75 (1.45-2.13) |  |
| **Coffee (cups/day)** |  |  |  |  |  |  |  |  |  |  |
| >2 | 141 | 245,846 | 1.00 (ref.) | 187 | 245,789 | 1.33 (1.07-1.65) | 263 | 250,349 | 1.96 (1.60-2.40) | 0.72 |
| 1 to ≤2 | 200 | 318,200 | 1.10 (0.88-1.36) | 255 | 320,727 | 1.39 (1.13-1.70) | 298 | 244,705 | 1.92 (1.58-2.33) |  |
| <1 | 127 | 240,629 | 0.99 (0.78-1.26) | 170 | 243,215 | 1.31 (1.05-1.64) | 336 | 314,053 | 2.07 (1.69-2.54) |  |
| **Tea (cups/day)** |  |  |  |  |  |  |  |  |  |  |
| ≥5 | 149 | 248,940 | 1.00 (ref.) | 160 | 250,934 | 1.07 (0.85-1.33) | 276 | 246,972 | 1.74 (1.43-2.13) | 0.05 |
| <5 | 132 | 239,193 | 0.92 (0.73-1.17) | 180 | 242,253 | 1.23 (0.99-1.53) | 353 | 319,989 | 2.03 (1.67-2.47) |  |
| <3 | 187 | 316,107 | 1.02 (0.82-1.27) | 272 | 316,074 | 1.49 (1.22-1.82) | 268 | 242,317 | 1.86 (1.53-2.25) |  |
| **Alcohol (times/week)** |  |  |  |  |  |  |  |  |  |  |
| <1 | 168 | 280,357 | 1.00 (ref.) | 226 | 282,699 | 1.34 (1.10-1.64) | 354 | 281,911 | 2.10 (1.75-2.52) | 0.46 |
| 1 to 2 | 130 | 216,524 | 1.08 (0.86-1.36) | 151 | 216,004 | 1.25 (1.00-1.56) | 211 | 215,143 | 1.75 (1.43-2.14) |  |
| ≥3 | 170 | 308,257 | 0.96 (0.77-1.19) | 233 | 311,647 | 1.30 (1.06-1.58) | 330 | 313,055 | 1.82 (1.51-2.20) |  |
| **IV-weighted dietary score** |  |  |  |  |  |  |  |  |  |  |
| -13.22 to <-2.88 | 160 | 291,208 | 1.00 (ref.) | 208 | 287,723 | 1.31 (1.07-1.62) | 330 | 290,522 | 2.06 (1.71-2.49) | 0.53 |
| -2.88 to <1.09 | 157 | 252,528 | 1.17 (0.94-1.45) | 198 | 256,972 | 1.44 (1.17-1.78) | 262 | 253,383 | 1.92 (1.58-2.34) |  |
| 1.09 to 13.02 | 120 | 196,571 | 1.17 (0.93-1.49) | 159 | 199,505 | 1.53 (1.23-1.91) | 222 | 200,380 | 2.13 (1.74-2.61) |  |

PRS, polygenic risk score; HR, hazard ratio; CI, confidence interval; WCRF, World Cancer Research Fund; IV, inverse variance. Low PRS: 316 to <454; intermediate PRS: 454 to <483; high PRS: 483 to ≤621. The HRs were estimated using Cox proportional hazard models with adjustment for first-degree family history of colorectal cancer, household income, smoking status, alcohol consumption (except when alcohol intake and dietary score are exposures), body mass index, and physical activity.

**Table S8.** Joint effects of dietary intake and polygenic risk score on subsite-specific colorectal cancer risks

| **Dietary factor** | **Low PRS** | | | **Intermediate PRS** | | | **High PRS** | | | **P_interaction_** |
| --- | --- | --- | --- | --- | --- | --- | --- | --- | --- | --- |
|  | **No. cases** | **Person-years** | **HR (95% CI)** | **No. cases** | **Person-years** | **HR (95% CI)** | **No. cases** | **Person-years** | **HR (95% CI)** |  |
| **Colon cancer** | | | | | | | | | | |
| **WCRF dietary score** |  |  |  |  |  |  |  |  |  |  |
| 2-3 | 336 | 812,923 | 1.00 (ref.) | 453 | 810,979 | 1.36 (1.19-1.56) | 684 | 806,692 | 2.06 (1.81-2.34) | 0.93 |
| 0-1 | 367 | 681,901 | 1.09 (0.94-1.26) | 493 | 681,155 | 1.44 (1.26-1.66) | 764 | 681,205 | 2.25 (1.98-2.56) |  |
| **Red meat (times/week)** |  |  |  |  |  |  |  |  |  |  |
| <2 | 327 | 748,267 | 1.00 (ref.) | 421 | 739,638 | 1.30 (1.12-1.50) | 652 | 737,155 | 2.03 (1.78-2.32) | 0.80 |
| 2 to <3 | 195 | 433,038 | 0.96 (0.80-1.14) | 293 | 434,943 | 1.43 (1.22-1.67) | 420 | 432,288 | 2.05 (1.78-2.37) |  |
| ≥3 | 186 | 324,027 | 1.16 (0.97-1.39) | 245 | 327,432 | 1.51 (1.28-1.78) | 388 | 328,690 | 2.37 (2.04-2.74) |  |
| **Processed meat (times/week)** |  |  |  |  |  |  |  |  |  |  |
| <1 | 247 | 573,384 | 1.00 (ref.) | 330 | 574,369 | 1.32 (1.12-1.56) | 500 | 568,769 | 2.03 (1.74-2.36) | 0.81 |
| 1 | 220 | 448,827 | 1.07 (0.89-1.28) | 303 | 450,863 | 1.46 (1.24-1.73) | 438 | 452,507 | 2.11 (1.80-2.47) |  |
| ≥2 | 241 | 482,937 | 1.06 (0.88-1.26) | 325 | 476,784 | 1.44 (1.22-1.70) | 523 | 476,817 | 2.32 (1.99-2.71) |  |
| **Poultry (times/week)** |  |  |  |  |  |  |  |  |  |  |
| ≥2 | 318 | 721,709 | 1.00 (ref.) | 443 | 728,130 | 1.38 (1.19-1.59) | 656 | 723,896 | 2.05 (1.79-2.34) | 0.90 |
| 1 | 286 | 551,619 | 1.06 (0.91-1.25) | 368 | 545,482 | 1.38 (1.19-1.60) | 571 | 546,613 | 2.14 (1.86-2.45) |  |
| <1 | 105 | 231,662 | 0.96 (0.77-1.20) | 148 | 228,237 | 1.37 (1.13-1.67) | 232 | 227,185 | 2.18 (1.84-2.58) |  |
| **Total fish (times/week)** |  |  |  |  |  |  |  |  |  |  |
| >2 | 187 | 424,526 | 1.00 (ref.) | 277 | 418,437 | 1.49 (1.24-1.80) | 395 | 416,769 | 2.15 (1.81-2.56) | 0.70 |
| >1 to ≤2 | 344 | 690,774 | 1.17 (0.98-1.40) | 465 | 693,560 | 1.57 (1.33-1.86) | 710 | 693,116 | 2.40 (2.04-2.82) |  |
| ≤1 | 177 | 390,096 | 1.18 (0.96-1.46) | 217 | 390,221 | 1.44 (1.19-1.76) | 355 | 388,637 | 2.37 (1.98-2.83) |  |
| **Milk (100 mL/day)** |  |  |  |  |  |  |  |  |  |  |
| ≥3 | 134 | 330,834 | 1.00 (ref.) | 192 | 328,379 | 1.43 (1.15-1.79) | 326 | 329,500 | 2.41 (1.97-2.95) | 0.07 |
| 2 to <3 | 310 | 596,161 | 1.30 (1.06-1.59) | 398 | 593,079 | 1.68 (1.38-2.04) | 549 | 592,738 | 2.32 (1.92-2.80) |  |
| <2 | 236 | 503,725 | 1.24 (1.00-1.54) | 336 | 504,997 | 1.76 (1.44-2.16) | 523 | 501,314 | 2.78 (2.29-3.36) |  |
| **Cheese (times/week)** |  |  |  |  |  |  |  |  |  |  |
| <2 | 276 | 594,359 | 1.00 (ref.) | 385 | 593,345 | 1.39 (1.19-1.62) | 592 | 592,832 | 2.41 (1.97-2.95) | 0.82 |
| 2 to 4 | 335 | 685,943 | 1.07 (0.91-1.26) | 431 | 682,791 | 1.38 (1.19-1.61) | 649 | 682,984 | 2.32 (1.92-2.80) |  |
| ≥5 | 87 | 193,473 | 1.02 (0.80-1.30) | 116 | 193,811 | 1.36 (1.09-1.69) | 188 | 190,580 | 2.78 (2.39-3.36) |  |
| **Total fruit (servings/day)** |  |  |  |  |  |  |  |  |  |  |
| ≥4 | 155 | 321,556 | 1.00 (ref.) | 214 | 317,908 | 1.39 (1.13-1.71) | 325 | 318,200 | 2.11 (1.74-2.56) | 0.22 |
| 2 to <4 | 310 | 682,784 | 0.97 (0.80-1.18) | 402 | 681,033 | 1.26 (1.05-1.52) | 669 | 676,394 | 2.12 (1.78-2.53) |  |
| <2 | 244 | 500,812 | 1.04 (0.85-1.28) | 342 | 502,921 | 1.46 (1.20-1.77) | 465 | 503,306 | 1.97 (1.64-2.37) |  |
| **Total vegetables (servings/day)** |  |  |  |  |  |  |  |  |  |  |
| ≥6 | 211 | 450,423 | 1.00 (ref.) | 257 | 445,940 | 1.23 (1.02-1.47) | 448 | 442,604 | 2.16 (1.84-2.55) | 0.28 |
| <6 | 235 | 515,188 | 1.00 (0.83-1.20) | 338 | 512,290 | 1.43 (1.20-1.70) | 500 | 513,701 | 2.11 (1.80-2.48) |  |
| <4 | 258 | 533,182 | 1.09 (0.91-1.31) | 357 | 537,844 | 1.49 (1.26-1.77) | 505 | 535,006 | 2.12 (1.80-2.49) |  |
| **Coffee (cups/day)** |  |  |  |  |  |  |  |  |  |  |
| >2 | 265 | 501,768 | 1.00 (ref.) | 321 | 496,918 | 1.22 (1.04-1.44) | 423 | 468,064 | 1.94 (1.67-2.25) | 0.46 |
| 1 to ≤2 | 250 | 581,794 | 0.80 (0.68-0.96) | 378 | 581,380 | 1.22 (1.04-1.42) | 446 | 441,784 | 1.81 (1.56-2.09) |  |
| <1 | 193 | 421,501 | 0.93 (0.77-1.12) | 261 | 423,437 | 1.25 (1.05-1.48) | 589 | 587,633 | 1.87 (1.60-2.19) |  |
| **Tea (cups/day)** |  |  |  |  |  |  |  |  |  |  |
| ≥5 | 207 | 467,205 | 1.00 (ref.) | 278 | 469,620 | 1.33 (1.11-1.59) | 505 | 494,939 | 2.02 (1.71-2.38) | 0.99 |
| <5 | 212 | 442,677 | 1.07 (0.88-1.29) | 284 | 438,057 | 1.44 (1.20-1.72) | 562 | 579,249 | 2.25 (1.91-2.65) |  |
| <3 | 289 | 594,604 | 1.13 (0.95-1.36) | 397 | 593,522 | 1.56 (1.32-1.84) | 391 | 423,267 | 2.35 (2.01-2.76) |  |
| **Alcohol (times/week)** |  |  |  |  |  |  |  |  |  |  |
| <1 | 191 | 418,909 | 1.00 (ref.) | 259 | 420,935 | 1.36 (1.12-1.63) | 420 | 419,048 | 2.20 (1.85-2.61) | 0.73 |
| 1 to 2 | 178 | 401,076 | 1.01 (0.82-1.24) | 228 | 399,874 | 1.28 (1.06-1.56) | 337 | 399,153 | 1.90 (1.59-2.28) |  |
| ≥3 | 340 | 686,270 | 1.03 (0.86-1.24) | 471 | 682,105 | 1.44 (1.21-1.70) | 701 | 680,988 | 2.14 (1.82-2.52) |  |
| **IV-weighted dietary score** |  |  |  |  |  |  |  |  |  |  |
| -13.22 to <-2.88 | 179 | 467,287 | 1.00 (ref.) | 264 | 461,715 | 1.48 (1.23-1.79) | 407 | 464,662 | 2.26 (1.90-2.70) | 0.56 |
| -2.88 to <1.09 | 240 | 466,751 | 1.33 (1.10-1.61) | 293 | 468,417 | 1.61 (1.34-1.94) | 463 | 462,160 | 2.59 (2.18-3.08) |  |
| 1.09 to 13.02 | 250 | 463,047 | 1.38 (1.13-1.67) | 332 | 462,858 | 1.83 (1.53-2.20) | 494 | 463,360 | 2.72 (2.29-3.23) |  |
| **Rectal cancer** | | | | | | | | | | |
| **WCRF dietary score** |  |  |  |  |  |  |  |  |  |  |
| 2-3 | 148 | 812,923 | 1.00 (ref.) | 198 | 810,979 | 1.37 (1.11-1.68) | 315 | 806,692 | 2.14 (1.78-2.59) | 0.40 |
| 0-1 | 177 | 681,901 | 1.07 (0.86-1.33) | 292 | 681,155 | 1.77 (1.45-2.15) | 406 | 681,205 | 2.49 (2.07-3.01) |  |
| **Red meat (times/week)** |  |  |  |  |  |  |  |  |  |  |
| <2 | 135 | 748,267 | 1.00 (ref.) | 214 | 739,638 | 1.60 (1.29-1.99) | 337 | 737,155 | 2.56 (2.10-3.13) | 0.36 |
| 2 to <3 | 107 | 433,038 | 1.24 (0.96-1.60) | 144 | 434,943 | 1.67 (1.32-2.12) | 211 | 432,288 | 2.46 (1.98-3.06) |  |
| ≥3 | 88 | 324,027 | 1.28 (0.98-1.68) | 137 | 327,432 | 1.99 (1.56-2.52) | 181 | 328,690 | 2.61 (2.09-3.26) |  |
| **Processed meat (times/week)** |  |  |  |  |  |  |  |  |  |  |
| <1 | 98 | 573,384 | 1.00 (ref.) | 151 | 574,369 | 1.53 (1.19-1.97) | 241 | 568,769 | 2.49 (1.97-3.15) | 0.77 |
| 1 | 99 | 448,827 | 1.13 (0.85-1.49) | 152 | 450,863 | 1.73 (1.34-2.23) | 211 | 452,507 | 2.39 (1.88-3.04) |  |
| ≥2 | 132 | 482,937 | 1.26 (0.97-1.64) | 192 | 476,784 | 1.87 (1.46-2.39) | 277 | 476,817 | 2.70 (2.14-3.41) |  |
| **Poultry (times/week)** |  |  |  |  |  |  |  |  |  |  |
| ≥2 | 145 | 721,709 | 1.00 (ref.) | 233 | 728,130 | 1.60 (1.30-1.97) | 347 | 723,896 | 2.40 (1.98-2.91) | 0.43 |
| 1 | 133 | 551,619 | 1.10 (0.87-1.39) | 201 | 545,482 | 1.67 (1.35-2.07) | 274 | 546,613 | 2.29 (1.87-2.80) |  |
| <1 | 52 | 231,662 | 1.07 (0.78-1.47) | 59 | 228,237 | 1.23 (0.91-1.67) | 107 | 227,185 | 2.26 (1.76-2.91) |  |
| **Total fish (times/week)** |  |  |  |  |  |  |  |  |  |  |
| >2 | 103 | 424,526 | 1.00 (ref.) | 128 | 418,437 | 1.26 (0.97-1.64) | 213 | 416,769 | 2.12 (1.67-2.68) | 0.51 |
| >1 to ≤2 | 151 | 690,774 | 0.92 (0.72-1.18) | 246 | 693,560 | 1.50 (1.19-1.89) | 356 | 693,116 | 2.18 (1.75-2.72) |  |
| ≤1 | 75 | 390,096 | 0.89 (0.66-1.19) | 120 | 390,221 | 1.41 (1.09-1.84) | 160 | 388,637 | 1.90 (1.48-2.44) |  |
| **Milk (100 mL/day)** |  |  |  |  |  |  |  |  |  |  |
| ≥3 | 80 | 330,834 | 1.00 (ref.) | 101 | 328,379 | 1.27 (0.95-1.70) | 156 | 329,500 | 1.97 (1.50-2.57) | 0.33 |
| 2 to <3 | 114 | 596,161 | 0.80 (0.60-1.07) | 199 | 593,079 | 1.42 (1.09-1.84) | 291 | 592,738 | 2.08 (1.62-2.66) |  |
| <2 | 125 | 503,725 | 1.10 (0.83-1.46) | 174 | 504,997 | 1.53 (1.17-1.99) | 246 | 501,314 | 2.19 (1.70-2.82) |  |
| **Cheese (times/week)** |  |  |  |  |  |  |  |  |  |  |
| <2 | 121 | 594,359 | 1.00 (ref.) | 173 | 593,345 | 1.43 (1.14-1.81) | 302 | 592,832 | 2.52 (2.04-3.11) | 0.08 |
| 2 to 4 | 158 | 685,943 | 1.09 (0.86-1.38) | 239 | 682,791 | 1.66 (1.33-2.07) | 299 | 682,984 | 2.08 (1.69-2.58) |  |
| ≥5 | 45 | 193,473 | 1.13 (0.80-1.59) | 67 | 193,811 | 1.67 (1.24-2.26) | 106 | 190,580 | 2.70 (2.08-3.51) |  |
| **Total fruit (servings/day)** |  |  |  |  |  |  |  |  |  |  |
| ≥4 | 79 | 321,556 | 1.00 (ref.) | 94 | 317,908 | 1.21 (0.90-1.63) | 131 | 318,200 | 1.69 (1.28-2.23) | 0.14 |
| 2 to <4 | 140 | 682,784 | 0.84 (0.64-1.11) | 215 | 681,033 | 1.29 (1.00-1.67) | 302 | 676,394 | 1.84 (1.44-2.36) |  |
| <2 | 110 | 500,812 | 0.85 (0.63-1.13) | 186 | 502,921 | 1.44 (1.10-1.87) | 297 | 503,306 | 2.28 (1.78-2.93) |  |
| **Total vegetables (servings/day)** |  |  |  |  |  |  |  |  |  |  |
| ≥6 | 121 | 450,423 | 1.00 (ref.) | 129 | 445,940 | 1.08 (0.84-1.39) | 218 | 442,604 | 1.84 (1.48-2.30) | **0.02** |
| <6 | 114 | 515,188 | 0.83 (0.65-1.08) | 178 | 512,290 | 1.31 (1.04-1.64) | 249 | 513,701 | 1.84 (1.48-2.29) |  |
| <4 | 92 | 533,182 | 0.65 (0.49-0.85) | 183 | 537,844 | 1.29 (1.02-1.62) | 254 | 535,006 | 1.80 (1.45-2.23) |  |
| **Coffee (cups/day)** |  |  |  |  |  |  |  |  |  |  |
| >2 | 104 | 501,768 | 1.00 (ref.) | 172 | 496,918 | 1.68 (1.31-2.14) | 206 | 468,064 | 2.43 (1.94-3.06) | 0.58 |
| 1 to ≤2 | 138 | 581,794 | 1.16 (0.90-1.49) | 200 | 581,380 | 1.68 (1.33-2.13) | 238 | 441,784 | 2.34 (1.87-2.94) |  |
| <1 | 88 | 421,501 | 1.12 (0.84-1.49) | 122 | 423,437 | 1.54 (1.19-2.01) | 284 | 587,633 | 2.57 (2.03-3.26) |  |
| **Tea (cups/day)** |  |  |  |  |  |  |  |  |  |  |
| ≥5 | 115 | 467,205 | 1.00 (ref.) | 138 | 469,620 | 1.19 (0.93-1.53) | 247 | 494,939 | 1.79 (1.42-2.24) | **0.03** |
| <5 | 79 | 442,677 | 0.71 (0.54-0.95) | 154 | 438,057 | 1.42 (1.11-1.80) | 277 | 579,249 | 2.18 (1.75-2.73) |  |
| <3 | 136 | 594,604 | 0.96 (0.75-1.23) | 203 | 593,522 | 1.43 (1.14-1.80) | 203 | 423,267 | 2.04 (1.64-2.53) |  |
| **Alcohol (times/week)** |  |  |  |  |  |  |  |  |  |  |
| <1 | 69 | 418,909 | 1.00 (ref.) | 114 | 420,935 | 1.65 (1.23-2.23) | 159 | 419,048 | 2.32 (1.75-3.07) | 0.86 |
| 1 to 2 | 85 | 401,076 | 1.23 (0.89-1.69) | 125 | 399,874 | 1.80 (1.34-2.42) | 174 | 399,153 | 2.53 (1.91-3.34) |  |
| ≥3 | 175 | 686,270 | 1.31 (0.99-1.74) | 256 | 682,105 | 1.94 (1.48-2.54) | 397 | 680,988 | 3.03 (2.34-3.92) |  |
| **IV-weighted dietary score** |  |  |  |  |  |  |  |  |  |  |
| -13.22 to <-2.88 | 100 | 467,287 | 1.00 (ref.) | 113 | 461,715 | 1.14 (0.87-1.49) | 185 | 464,662 | 1.86 (1.46-2.37) | 0.24 |
| -2.88 to <1.09 | 89 | 466,751 | 0.85 (0.64-1.13) | 154 | 468,417 | 1.47 (1.14-1.89) | 210 | 462,160 | 2.04 (1.61-2.59) |  |
| 1.09 to 13.02 | 120 | 463,047 | 1.08 (0.83-1.41) | 191 | 462,858 | 1.73 (1.36-2.21) | 269 | 463,360 | 2.44 (1.94-3.08) |  |

PRS, polygenic risk score; HR, hazard ratio; CI, confidence interval; WCRF, World Cancer Research Fund; IV, inverse variance. Low PRS: 316 to <454; intermediate PRS: 454 to <483; high PRS: 483 to ≤621. The HRs were estimated using Cox proportional hazard models with adjustment for sex, first-degree family history of colorectal cancer, household income, smoking status, alcohol consumption (except when alcohol intake and dietary score are exposures), body mass index, and physical activity.

**Table S9.** Sex-specific estimates of cumulative risk of developing colorectal cancer at age 80 years and associations between dietary intake and colorectal cancer according to polygenic risk score categories

| **Dietary factor** | **Low PRS** | | **Intermediate PRS** | | **High PRS** | |
| --- | --- | --- | --- | --- | --- | --- |
|  | **CR (%)** | **HR (95% CI)** | **CR (%)** | **HR (95% CI)** | **CR (%)** | **HR (95% CI)** |
| **Men** | | | | | | |
| **WCRF dietary score** |  |  |  |  |  |  |
| 2-3 | 2.62 | 1.00 (ref.) | 3.40 | 1.00 (ref.) | 5.58 | 1.00 (ref.) |
| 0-1 | 3.02 | 1.15 (0.97-1.37) | 4.25 | **1.25 (1.08-1.44)** | 6.39 | **1.15 (1.03-1.29)** |
| **Red meat (times/week)** |  |  |  |  |  |  |
| <2 | 2.72 | 1.00 (ref.) | 3.61 | 1.00 (ref.) | 5.76 | 1.00 (ref.) |
| 2 to <3 | 2.69 | 0.99 (0.81-1.21) | 3.79 | 1.05 (0.89-1.24) | 5.93 | 1.03 (0.90-1.18) |
| ≥3 | 3.22 | 1.19 (0.97-1.45) | 4.55 | **1.27 (1.08-1.49)** | 6.62 | **1.15 (1.01-1.32)** |
| **Processed meat (times/week)** |  |  |  |  |  |  |
| <1 | 2.49 | 1.00 (ref.) | 3.42 | 1.00 (ref.) | 5.57 | 1.00 (ref.) |
| 1 | 2.82 | 1.14 (0.90-1.43) | 3.81 | 1.12 (0.92-1.35) | 5.96 | 1.07 (0.92-1.25) |
| ≥2 | 3.05 | 1.23 (0.99-1.52) | 4.27 | **1.26 (1.05-1.50)** | 6.35 | 1.14 (0.99-1.32) |
| **Poultry (times/week)** |  |  |  |  |  |  |
| <1 | 2.57 | 1.00 (ref.) | 3.66 | 1.00 (ref.) | 5.39 | 1.00 (ref.) |
| 1 | 3.03 | 1.18 (0.92-1.53) | 3.95 | 1.08 (0.88-1.33) | 6.13 | 1.14 (0.96-1.36) |
| ≥2 | 2.78 | 1.08 (0.84-1.40) | 3.95 | 1.08 (0.88-1.33) | 6.18 | 1.15 (0.97-1.37) |
| **Total fish (times/week)** |  |  |  |  |  |  |
| ≤1 | 2.85 | 1.00 (ref.) | 3.6 | 1.00 (ref.) | 5.88 | 1.00 (ref.) |
| >1 to ≤2 | 2.9 | 1.02 (0.83-1.25) | 4.19 | 1.17 (0.98-1.39) | 6.23 | 1.06 (0.93-1.22) |
| >2 | 2.73 | 0.96 (0.76-1.21) | 3.7 | 1.03 (0.85-1.25) | 5.84 | 0.99 (0.85-1.16) |
| **Milk (100 mL/day)** |  |  |  |  |  |  |
| <2 | 3.19 | 1.00 (ref.) | 4.09 | 1.00 (ref.) | 6.65 | 1.00 (ref.) |
| 2 to <3 | 2.86 | 0.89 (0.74-1.08) | 3.96 | 0.97 (0.83-1.14) | 5.80 | **0.87 (0.77-0.99)** |
| ≥3 | 2.47 | **0.77 (0.61-0.97)** | 3.67 | 0.90 (0.74-1.08) | 5.69 | **0.86 (0.74-0.99)** |
| **Cheese (times/week)** |  |  |  |  |  |  |
| <2 | 2.83 | 1.00 (ref.) | 3.87 | 1.00 (ref.) | 6.53 | 1.00 (ref.) |
| 2 to 4 | 2.89 | 1.03 (0.86-1.23) | 3.91 | 1.01 (0.87-1.17) | 5.56 | **0.85 (0.75-0.96)** |
| ≥5 | 2.98 | 1.06 (0.82-1.38) | 3.91 | 1.01 (0.81-1.25) | 6.39 | 0.98 (0.82-1.16) |
| **Total fruit (servings/day)** |  |  |  |  |  |  |
| <2 | 2.91 | 1.00 (ref.) | 4.33 | 1.00 (ref.) | 6.43 | 1.00 (ref.) |
| 2 to <4 | 2.57 | 0.88 (0.73-1.06) | 3.50 | **0.80 (0.69-0.94)** | 5.87 | 0.91 (0.81-1.03) |
| ≥4 | 3.33 | 1.14 (0.91-1.43) | 3.96 | 0.91 (0.75-1.10) | 5.59 | 0.86 (0.74-1.01) |
| **Total vegetables (servings/day)** |  |  |  |  |  |  |
| <4 | 2.78 | 1.00 (ref.) | 4.09 | 1.00 (ref.) | 5.94 | 1.00 (ref.) |
| 4 to <6 | 2.88 | 1.04 (0.85-1.27) | 4.09 | 1.00 (0.86-1.17) | 5.99 | 1.01 (0.89-1.15) |
| ≥6 | 2.89 | 1.04 (0.85-1.28) | 3.32 | **0.81 (0.68-0.97)** | 6.22 | 1.05 (0.91-1.20) |
| **Coffee (cups/day)** |  |  |  |  |  |  |
| <1 | 3.09 | 1.00 (ref.) | 3.9 | 1.00 (ref.) | 5.98 | 1.00 (ref.) |
| 1 to ≤2 | 2.39 | **0.77 (0.62-0.96)** | 3.83 | 0.98 (0.82-1.17) | 5.84 | 0.97 (0.85-1.12) |
| >2 | 3.18 | 1.03 (0.84-1.26) | 4.00 | 1.02 (0.86-1.22) | 6.26 | 1.05 (0.91-1.21) |
| **Tea (cups/day)** |  |  |  |  |  |  |
| <3 | 3.07 | 1.00 (ref.) | 3.93 | 1.00 (ref.) | 6.5 | 1.00 (ref.) |
| 3 to <5 | 2.63 | 0.85 (0.70-1.04) | 4.08 | 1.04 (0.88-1.23) | 6.11 | 0.94 (0.82-1.07) |
| ≥5 | 2.76 | 0.90 (0.74-1.09) | 3.73 | 0.95 (0.80-1.12) | 5.37 | **0.82 (0.72-0.94)** |
| **Alcohol (times/week)** |  |  |  |  |  |  |
| <1 | 2.32 | 1.00 (ref.) | 3.47 | 1.00 (ref.) | 5.38 | 1.00 (ref.) |
| 1 to 2 | 2.65 | 1.15 (0.88-1.50) | 3.70 | 1.07 (0.86-1.32) | 5.55 | 1.03 (0.87-1..23) |
| ≥3 | 3.14 | **1.36 (1.08-1.72)** | 4.2 | **1.22 (1.01-1.47)** | 6.55 | **1.23 (1.05-1.43)** |
| **IV-weighted dietary score** |  |  |  |  |  |  |
| -13.22 to <-2.88 | 2.37 | 1.00 (ref.) | 3.10 | 1.00 (ref.) | 4.84 | 1.00 (ref.) |
| -2.88 to <1.09 | 2.81 | 1.19 (0.94-1.50) | 3.77 | 1.22 (1.00-1.48) | 6.37 | **1.33 (1.14-1.55)** |
| 1.09 to 13.02 | 3.34 | **1.42 (1.14-1.77)** | 4.54 | **1.47 (1.23-1.77)** | 6.69 | **1.40 (1.20-1.62)** |
| **Women** | | | | | | |
| **WCRF dietary score** |  |  |  |  |  |  |
| 2-3 | 1.97 | 1.00 (ref.) | 2.88 | 1.00 (ref.) | 3.79 | 1.00 (ref.) |
| 0-1 | 2.13 | 1.08 (0.89-1.31) | 2.86 | 0.99 (0.84-1.18) | 3.82 | 1.01 (0.87-1.16) |
| **Red meat (times/week)** |  |  |  |  |  |  |
| <2 | 1.88 | 1.00 (ref.) | 2.75 | 1.00 (ref.) | 3.82 | 1.00 (ref.) |
| 2 to <3 | 2.11 | 1.12 (0.91-1.39) | 3.09 | 1.13 (0.94-1.35) | 3.62 | 0.95 (0.81-1.11) |
| ≥3 | 2.28 | 1.21 (0.96-1.53) | 2.91 | 1.06 (0.86-1.31) | 4.04 | 1.06 (0.89-1.25) |
| **Processed meat (times/week)** |  |  |  |  |  |  |
| <1 | 1.93 | 1.00 (ref.) | 2.79 | 1.00 (ref.) | 3.84 | 1.00 (ref.) |
| 1 | 2.11 | 1.10 (0.89-1.35) | 3.18 | 1.14 (0.95-1.37) | 3.6 | 0.94 (0.80-1.09) |
| ≥2 | 2.12 | 1.10 (0.87-1.39) | 2.62 | 0.94 (0.75-1.16) | 4.04 | 1.05 (0.89-1.25) |
| **Poultry (times/week)** |  |  |  |  |  |  |
| <1 | 2.11 | 1.00 (ref.) | 2.56 | 1.00 (ref.) | 4.54 | 1.00 (ref.) |
| 1 | 2.04 | 0.97 (0.74-1.27) | 2.94 | 1.15 (0.90-1.47) | 3.80 | 0.83 (0.69-1.01) |
| ≥2 | 1.98 | 0.94 (0.72-1.22) | 2.93 | 1.15 (0.90-1.46) | 3.55 | **0.78 (0.65-0.93)** |
| **Total fish (times/week)** |  |  |  |  |  |  |
| ≤1 | 2.17 | 1.00 (ref.) | 3.04 | 1.00 (ref.) | 3.72 | 1.00 (ref.) |
| >1 to ≤2 | 2.05 | 0.95 (0.75-1.19) | 2.86 | 0.94 (0.77-1.15) | 3.99 | 1.07 (0.91-1.27) |
| >2 | 1.86 | 0.86 (0.66-1.10) | 2.76 | 0.91 (0.73-1.13) | 3.61 | 0.97 (0.81-1.17) |
| **Milk (100 mL/day)** |  |  |  |  |  |  |
| <2 | 2.08 | 1.00 (ref.) | 3.27 | 1.00 (ref.) | 4.01 | 1.00 (ref.) |
| 2 to <3 | 2.04 | 0.98 (0.80-1.22) | 2.94 | 0.90 (0.75-1.08) | 3.61 | 0.89 (0.76-1.04) |
| ≥3 | 1.96 | 0.94 (0.73-1.22) | 2.3 | **0.70 (0.56-0.89)** | 3.81 | 0.94 (0.79-1.13) |
| **Cheese (times/week)** |  |  |  |  |  |  |
| <2 | 1.87 | 1.00 (ref.) | 2.72 | 1.00 (ref.) | 3.72 | 1.00 (ref.) |
| 2 to 4 | 2.18 | 1.17 (0.96-1.43) | 2.96 | 1.09 (0.92-1.29) | 3.76 | 1.01 (0.88-1.17) |
| ≥5 | 2.01 | 1.08 (0.80-1.47) | 2.89 | 1.06 (0.81-1.39) | 4.24 | 1.15 (0.93-1.42) |
| **Total fruit (servings/day)** |  |  |  |  |  |  |
| <2 | 2.08 | 1.00 (ref.) | 2.92 | 1.00 (ref.) | 3.45 | 1.00 (ref.) |
| 2 to <4 | 2.08 | 1.00 (0.80-1.26) | 2.91 | 0.99 (0.82-1.21) | 3.94 | 1.15 (0.97-1.36) |
| ≥4 | 1.85 | 0.89 (0.69-1.16) | 2.77 | 0.95 (0.75-1.19) | 3.91 | 1.14 (0.94-1.37) |
| **Total vegetables (servings/day)** |  |  |  |  |  |  |
| <4 | 1.98 | 1.00 (ref.) | 3.11 | 1.00 (ref.) | 3.77 | 1.00 (ref.) |
| 4 to <6 | 1.90 | 0.96 (0.76-1.21) | 2.91 | 0.94 (0.77-1.13) | 3.84 | 1.02 (0.87-1.20) |
| ≥6 | 2.17 | 1.10 (0.88-1.38) | 2.63 | 0.85 (0.69-1.03) | 3.78 | 1.00 (0.85-1.19) |
| **Coffee (cups/day)** |  |  |  |  |  |  |
| <1 | 1.94 | 1.00 (ref.) | 2.84 | 1.00 (ref.) | 3.96 | 1.00 (ref.) |
| 1 to ≤2 | 2.13 | 1.09 (0.87-1.37) | 2.96 | 1.04 (0.86-1.27) | 3.71 | 0.94 (0.80-1.10) |
| >2 | 1.96 | 1.01 (0.79-1.28) | 2.82 | 0.99 (0.81-1.23) | 3.79 | 0.96 (0.81-1.13) |
| **Tea (cups/day)** |  |  |  |  |  |  |
| <3 | 2.10 | 1.00 (ref.) | 3.34 | 1.00 (ref.) | 3.76 | 1.00 (ref.) |
| 3 to <5 | 1.89 | 0.90 (0.72-1.12) | 2.76 | **0.82 (0.68-0.99)** | 4.13 | 1.10 (0.94-1.29) |
| ≥5 | 2.06 | 0.98 (0.79-1.22) | 2.41 | 0.72 (0.59-0.87) | 3.55 | 0.94 (0.80-1.11) |
| **Alcohol (times/week)** |  |  |  |  |  |  |
| <1 | 2.00 | 1.00 (ref.) | 2.97 | 1.00 (ref.) | 4.19 | 1.00 (ref.) |
| 1 to 2 | 2.17 | 1.09 (0.86-1.37) | 2.79 | 0.94 (0.76-1.16) | 3.48 | **0.83 (0.70-0.98)** |
| ≥3 | 1.94 | 0.97 (0.78-1.21) | 2.84 | 0.96 (0.79-1.16) | 3.66 | 0.87 (0.74-1.02) |
| **IV-weighted dietary score** |  |  |  |  |  |  |
| -13.22 to <-2.88 | 1.87 | 1.00 (ref.) | 2.70 | 1.00 (ref.) | 3.82 | 1.00 (ref.) |
| -2.88 to <1.09 | 2.18 | 1.17 (0.94-1.46) | 2.95 | 1.10 (0.90-1.33) | 3.57 | 0.93 (0.79-1.10) |
| 1.09 to 13.02 | 2.20 | 1.18 (0.93-1.50) | 3.12 | 1.16 (0.94-1.43) | 3.93 | 1.03 (0.86-1.22) |

PRS, polygenic risk score; CR, cumulative risk; HR, hazard ratio; CI, confidence interval; WCRF, World Cancer Research Fund; IV, inverse variance. Low PRS: 316 to <454; intermediate PRS: 454 to <483; high PRS: 483 to ≤621. The HRs were estimated using Cox proportional hazard models with adjustment for first-degree family history of colorectal cancer, household income, smoking status, alcohol consumption (except when alcohol intake and dietary score are exposures), body mass index, and physical activity.

**Table S10.** Subsite-specific estimates of cumulative risk of developing colorectal cancer at age 80 years and associations between dietary intake and colorectal cancer according to polygenic risk score categories

| **Dietary factor** | **Low PRS** | | **Intermediate PRS** | | **High PRS** | |
| --- | --- | --- | --- | --- | --- | --- |
|  | **CR (%)** | **HR (95% CI)** | **CR (%)** | **HR (95% CI)** | **CR (%)** | **HR (95% CI)** |
| **Colon cancer** | | | | | | |
| **WCRF dietary score** |  |  |  |  |  |  |
| 2-3 | 1.55 | 1.00 (ref.) | 2.14 | 1.00 (ref.) | 3.18 | 1.00 (ref.) |
| 0-1 | 1.76 | 1.14 (0.97-1.32) | 2.24 | 1.05 (0.92-1.20) | 3.44 | 1.08 (0.97-1.20) |
| **Red meat (times/week)** |  |  |  |  |  |  |
| <2 | 1.59 | 1.00 (ref.) | 2.06 | 1.00 (ref.) | 3.16 | 1.00 (ref.) |
| 2 to <3 | 1.53 | 0.96 (0.80-1.15) | 2.25 | 1.09 (0.94-1.27) | 3.22 | 1.02 (0.90-1.15) |
| ≥3 | 1.85 | 1.16 (0.97-1.40) | 2.37 | 1.15 (0.98-1.35) | 3.70 | **1.17 (1.03-1.33)** |
| **Processed meat (times/week)** |  |  |  |  |  |  |
| <1 | 1.55 | 1.00 (ref.) | 2.08 | 1.00 (ref.) | 3.13 | 1.00 (ref.) |
| 1 | 1.68 | 1.09 (0.90-1.31) | 2.27 | 1.09 (0.93-1.28) | 3.25 | 1.04 (0.91-1.18) |
| ≥2 | 1.7 | 1.10 (0.91-1.33) | 2.22 | 1.07 (0.91-1.25) | 3.55 | 1.14 (1.00-1.29) |
| **Poultry (times/week)** |  |  |  |  |  |  |
| <1 | 1.55 | 1.00 (ref.) | 2.17 | 1.00 (ref.) | 3.43 | 1.00 (ref.) |
| 1 | 1.71 | 1.10 (0.88-1.38) | 2.17 | 1.00 (0.83-1.21) | 3.37 | 0.98 (0.84-1.15) |
| ≥2 | 1.60 | 1.03 (0.83-1.29) | 2.21 | 1.02 (0.85-1.23) | 3.20 | 0.93 (0.80-1.08) |
| **Total fish (times/week)** |  |  |  |  |  |  |
| ≤1 | 1.73 | 1.00 (ref.) | 2.11 | 1.00 (ref.) | 3.34 | 1.00 (ref.) |
| >1 to ≤2 | 1.7 | 0.98 (0.82-1.18) | 2.26 | 1.07 (0.91-1.26) | 3.42 | 1.02 (0.90-1.17) |
| >2 | 1.46 | 0.84 (0.68-1.03) | 2.15 | 1.02 (0.85-1.22) | 3.07 | 0.92 (0.79-1.06) |
| **Milk (100 mL/day)** |  |  |  |  |  |  |
| <2 | 1.69 | 1.00 (ref.) | 2.39 | 1.00 (ref.) | 3.67 | 1.00 (ref.) |
| 2 to <3 | 1.77 | 1.05 (0.88-1.24) | 2.25 | 0.94 (0.81-1.09) | 3.09 | **0.84 (0.75-0.95)** |
| ≥3 | 1.37 | 0.81 (0.65-1.00) | 1.92 | 0.80 (0.67-0.96) | 3.22 | 0.87 (0.76-1.01) |
| **Cheese (times/week)** |  |  |  |  |  |  |
| <2 | 1.58 | 1.00 (ref.) | 2.21 | 1.00 (ref.) | 3.35 | 1.00 (ref.) |
| 2 to 4 | 1.72 | 1.09 (0.93-1.28) | 2.17 | 0.98 (0.86-1.13) | 3.24 | 0.97 (0.86-1.08) |
| ≥5 | 1.65 | 1.05 (0.82-1.34) | 2.14 | 0.97 (0.78-1.19) | 3.46 | 1.04 (0.88-1.22) |
| **Total fruit (servings/day)** |  |  |  |  |  |  |
| <2 | 1.72 | 1.00 (ref.) | 2.46 | 1.00 (ref.) | 3.14 | 1.00 (ref.) |
| 2 to <4 | 1.58 | 0.92 (0.77-1.09) | 2.04 | 0.86 (0.74-1.00) | 3.39 | 1.08 (0.96-1.22) |
| ≥4 | 1.62 | 0.94 (0.77-1.16) | 2.25 | 0.95 (0.80-1.14) | 3.38 | 1.08 (0.93-1.25) |
| **Total vegetables (servings/day)** |  |  |  |  |  |  |
| <4 | 1.74 | 1.00 (ref.) | 2.34 | 1.00 (ref.) | 3.27 | 1.00 (ref.) |
| 4 to <6 | 1.58 | 0.91 (0.76-1.08) | 2.24 | 0.96 (0.82-1.11) | 3.29 | 1.00 (0.89-1.14) |
| ≥6 | 1.58 | 0.91 (0.76-1.09) | 1.94 | **0.83 (0.70-0.97)** | 3.36 | 1.03 (0.90-1.17) |
| **Coffee (cups/day)** |  |  |  |  |  |  |
| <1 | 1.68 | 1.00 (ref.) | 2.25 | 1.00 (ref.) | 3.29 | 1.00 (ref.) |
| 1 to ≤2 | 1.45 | 0.86 (0.71-1.04) | 2.17 | 0.96 (0.82-1.13) | 3.21 | 0.98 (0.86-1.11) |
| >2 | 1.82 | 1.08 (0.90-1.30) | 2.18 | 0.97 (0.82-1.14) | 3.42 | 1.04 (0.91-1.19) |
| **Tea (cups/day)** |  |  |  |  |  |  |
| <3 | 1.73 | 1.00 (ref.) | 2.36 | 1.00 (ref.) | 3.49 | 1.00 (ref.) |
| 3 to <5 | 1.63 | 0.94 (0.79-1.12) | 2.16 | 0.92 (0.79-1.07) | 3.36 | 0.96 (0.85-1.09) |
| ≥5 | 1.53 | 0.88 (0.74-1.06) | 2.01 | **0.85 (0.73-0.99)** | 3.01 | **0.86 (0.76-0.97)** |
| **Alcohol (times/week)** |  |  |  |  |  |  |
| <1 | 1.55 | 1.00 (ref.) | 2.17 | 1.00 (ref.) | 3.53 | 1.00 (ref.) |
| 1 to 2 | 1.62 | 1.05 (0.85-1.29) | 2.06 | 0.95 (0.79-1.14) | 3.00 | **0.85 (0.73-0.98)** |
| ≥3 | 1.71 | 1.11 (0.92-1.33) | 2.28 | 1.05 (0.90-1.23) | 3.35 | 0.95 (0.83-1.08) |
| **IV-weighted dietary score** |  |  |  |  |  |  |
| -13.22 to <-2.88 | 1.33 | 1.00 (ref.) | 1.99 | 1.00 (ref.) | 3.01 | 1.00 (ref.) |
| -2.88 to <1.09 | 1.80 | **1.35 (1.12-1.65)** | 2.16 | 1.09 (0.92-1.28) | 3.42 | 1.14 (0.99-1.30) |
| 1.09 to 13.02 | 1.89 | **1.43 (1.18-1.74)** | 2.44 | **1.23 (1.05-1.46)** | 3.55 | **1.18 (1.03-1.35)** |
| **Rectal cancer** | | | | | | |
| **WCRF dietary score** |  |  |  |  |  |  |
| 2-3 | 0.75 | 1.00 (ref.) | 1.02 | 1.00 (ref.) | 1.52 | 1.00 (ref.) |
| 0-1 | 0.82 | 1.10 (0.88-1.38) | 1.36 | **1.34 (1.11-1.61)** | 1.70 | 1.12 (0.96-1.30) |
| **Red meat (times/week)** |  |  |  |  |  |  |
| <2 | 0.69 | 1.00 (ref.) | 1.1 | 1.00 (ref.) | 1.63 | 1.00 (ref.) |
| 2 to <3 | 0.86 | 1.25 (0.97-1.61) | 1.17 | 1.06 (0.86-1.31) | 1.55 | 0.95 (0.80-1.13) |
| ≥3 | 0.89 | 1.30 (0.99-1.71) | 1.39 | **1.26 (1.02-1.57)** | 1.64 | 1.00 (0.83-1.20) |
| **Processed meat (times/week)** |  |  |  |  |  |  |
| <1 | 0.68 | 1.00 (ref.) | 1.04 | 1.00 (ref.) | 1.63 | 1.00 (ref.) |
| 1 | 0.78 | 1.15 (0.87-1.53) | 1.2 | 1.16 (0.92-1.46) | 1.52 | 0.93 (0.77-1.12) |
| ≥2 | 0.89 | 1.32 (1.00-1.74) | 1.33 | **1.28 (1.03-1.60)** | 1.67 | 1.02 (0.85-1.23) |
| **Poultry (times/week)** |  |  |  |  |  |  |
| <1 | 0.79 | 1.00 (ref.) | 0.92 | 1.00 (ref.) | 1.58 | 1.00 (ref.) |
| 1 | 0.82 | 1.03 (0.74-1.42) | 1.26 | **1.37 (1.03-1.84)** | 1.58 | 1.00 (0.80-1.25) |
| ≥2 | 0.76 | 0.96 (0.70-1.32) | 1.21 | 1.32 (0.99-1.75) | 1.64 | 1.04 (0.83-1.29) |
| **Total fish (times/week)** |  |  |  |  |  |  |
| ≤1 | 0.76 | 1.00 (ref.) | 1.19 | 1.00 (ref.) | 1.44 | 1.00 (ref.) |
| >1 to ≤2 | 0.77 | 1.01 (0.76-1.33) | 1.26 | 1.06 (0.85-1.32) | 1.58 | 1.16 (0.96-1.41) |
| >2 | 0.83 | 1.09 (0.80-1.47) | 1.06 | 0.88 (0.69-1.14) | 1.64 | 1.14 (0.92-1.40) |
| **Milk (100 mL/day)** |  |  |  |  |  |  |
| <2 | 0.93 | 1.00 (ref.) | 1.29 | 1.00 (ref.) | 1.66 | 1.00 (ref.) |
| 2 to <3 | 0.67 | **0.72 (0.56-0.93)** | 1.19 | 0.92 (0.75-1.13) | 1.60 | 0.96 (0.81-1.14) |
| ≥3 | 0.83 | 0.89 (0.67-1.18) | 1.06 | 0.83 (0.65-1.06) | 1.51 | 0.91 (0.74-1.11) |
| **Cheese (times/week)** |  |  |  |  |  |  |
| <2 | 0.75 | 1.00 (ref.) | 1.06 | 1.00 (ref.) | 1.73 | 1.00 (ref.) |
| 2 to 4 | 0.81 | 1.09 (0.86-1.39) | 1.25 | 1.17 (0.96-1.43) | 1.42 | **0.82 (0.70-0.96)** |
| ≥5 | 0.84 | 1.13 (0.80-1.59) | 1.26 | 1.18 (0.89-1.57) | 1.84 | 1.06 (0.85-1.33) |
| **Total fruit (servings/day)** |  |  |  |  |  |  |
| <2 | 0.76 | 1.00 (ref.) | 1.30 | 1.00 (ref.) | 1.85 | 1.00 (ref.) |
| 2 to <4 | 0.75 | 0.98 (0.76-1.27) | 1.16 | 0.89 (0.73-1.08) | 1.52 | **0.82 (0.70-0.97)** |
| ≥4 | 0.89 | 1.16 (0.86-1.56) | 1.07 | 0.82 (0.64-1.06) | 1.41 | **0.76 (0.62-0.94)** |
| **Total vegetables (servings/day)** |  |  |  |  |  |  |
| <4 | 0.62 | 1.00 (ref.) | 1.24 | 1.00 (ref.) | 1.56 | 1.00 (ref.) |
| 4 to <6 | 0.80 | 1.27 (0.97-1.68) | 1.25 | 1.00 (0.81-1.23) | 1.62 | 1.04 (0.87-1.24) |
| ≥6 | 0.96 | **1.54 (1.17-2.03)** | 1.03 | 0.82 (0.66-1.04) | 1.63 | 1.04 (0.87-1.25) |
| **Coffee (cups/day)** |  |  |  |  |  |  |
| <1 | 0.81 | 1.00 (ref.) | 1.11 | 1.00 (ref.) | 1.70 | 1.00 (ref.) |
| 1 to ≤2 | 0.82 | 1.01 (0.77-1.32) | 1.21 | 1.09 (0.87-1.37) | 1.56 | 0.92 (0.76-1.10) |
| >2 | 0.73 | 0.89 (0.67-1.19) | 1.22 | 1.10 (0.87-1.39) | 1.59 | 0.94 (0.78-1.13) |
| **Tea (cups/day)** |  |  |  |  |  |  |
| <3 | 0.85 | 1.00 (ref.) | 1.26 | 1.00 (ref.) | 1.63 | 1.00 (ref.) |
| 3 to <5 | 0.62 | **0.73 (0.55-0.97)** | 1.24 | 0.99 (0.80-1.22) | 1.76 | 1.08 (0.91-1.29) |
| ≥5 | 0.88 | 1.04 (0.81-1.33) | 1.05 | 0.83 (0.67-1.03) | 1.44 | 0.88 (0.74-1.06) |
| **Alcohol (times/week)** |  |  |  |  |  |  |
| <1 | 0.64 | 1.00 (ref.) | 1.07 | 1.00 (ref.) | 1.39 | 1.00 (ref.) |
| 1 to 2 | 0.80 | 1.25 (0.90-1.72) | 1.18 | 1.11 (0.86-1.43) | 1.50 | 1.07 (0.86-1.33) |
| ≥3 | 0.85 | 1.33 (1.00-1.79) | 1.26 | 1.18 (0.94-1.49) | 1.79 | **1.29 (1.06-1.56)** |
| **IV-weighted dietary score** |  |  |  |  |  |  |
| -13.22 to <-2.88 | 0.80 | 1.00 (ref.) | 0.91 | 1.00 (ref.) | 1.40 | 1.00 (ref.) |
| -2.88 to <1.09 | 0.69 | 0.86 (0.65-1.15) | 1.19 | **1.31 (1.03-1.67)** | 1.52 | 1.08 (0.89-1.32) |
| 1.09 to 13.02 | 0.89 | 1.11 (0.85-1.45) | 1.43 | **1.57 (1.24-1.99)** | 1.79 | **1.27 (1.05-1.53)** |

PRS, polygenic risk score; CR, cumulative risk; HR, hazard ratio; CI, confidence interval; WCRF, World Cancer Research Fund; IV, inverse variance. Low PRS: 316 to <454; intermediate PRS: 454 to <483; high PRS: 483 to ≤621. The HRs were estimated using Cox proportional hazard models with adjustment for sex, first-degree family history of colorectal cancer, household income, smoking status, alcohol consumption (except when alcohol intake and dietary score are exposures), body mass index, and physical activity.

**Table S11.** Attributable fraction of colorectal cancer due to dietary and genetic factors

| **Subgroup** | **WCRF dietary score** | **IV-weighted dietary score** | **PRS** |
| --- | --- | --- | --- |
| Total | 5.17 | 11.24 | 33.63 |
| Men | 9.60 | 19.28 | 37.29 |
| Women | 0.67 | 3.81 | 28.80 |
| Colon cancer | 3.93 | 11.24 | 32.28 |
| Rectal cancer | 8.18 | 10.99 | 36.84 |

Data are presented as percentage. WCRF, World Cancer Research Fund; IV, inverse-variance; PRS, polygenic risk score.
