# Supplementary material for: Assessments of dietary intake and polygenic risk score in associations with colorectal cancer risk: evidence from the UK Biobank
Source: BMC Cancer. 2023 Oct 18;23:993. doi: 10.1186/s12885-023-11482-1 (PMC10583398; doi:10.1186/s12885-023-11482-1)
Supplement: Supplementary file 2 — Additional file 2. [file 12885_2023_11482_MOESM2_ESM.docx]

**Assessments of dietary intake and polygenic risk score in associations with colorectal cancer risk: evidence from the UK Biobank**

**Additional file 2: Supplementary Figures**

**Figure S1.** Hazard ratios for colorectal cancer risk in (A) men and (B) women by deciles of each polygenic risk score

PRS, polygenic risk score; HR, hazard ratio; CI, confidence interval. HRs were estimated using Cox proportional hazard models with adjustment for first-degree family history of colorectal cancer.

**Figure S2.** Hazard ratios for (A) colon and (B) rectal cancer risks by deciles of each polygenic risk score

PRS, polygenic risk score; HR, hazard ratio; CI, confidence interval. HRs were estimated using Cox proportional hazard models with adjustment for sex and first-degree family history of colorectal cancer.

Canonical pathway


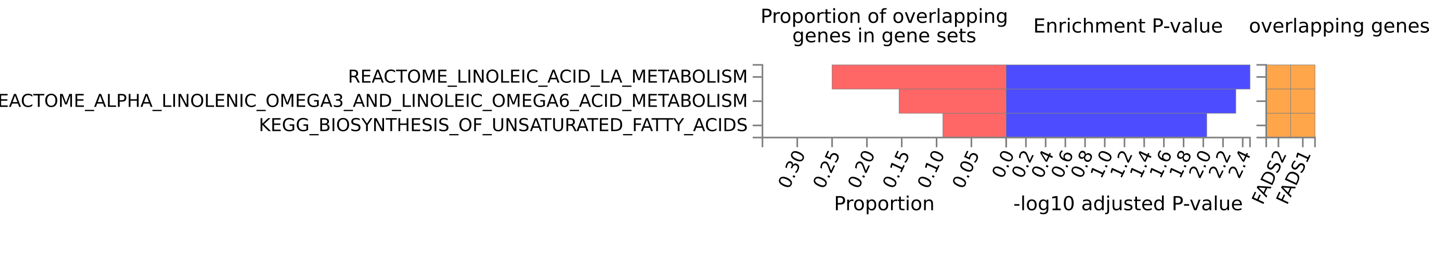


KEGG pathway


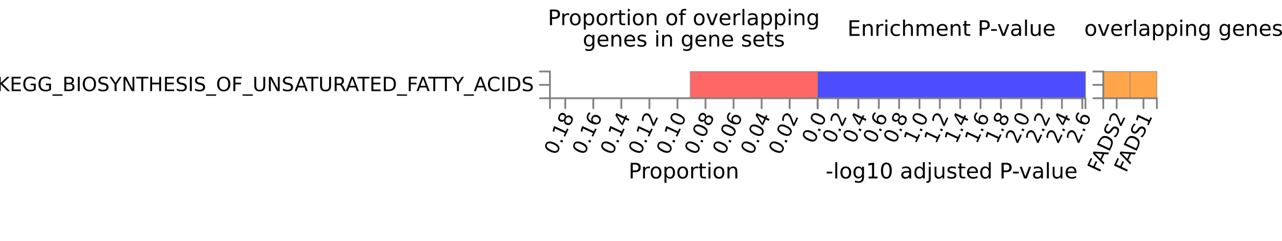


Reactome pathway


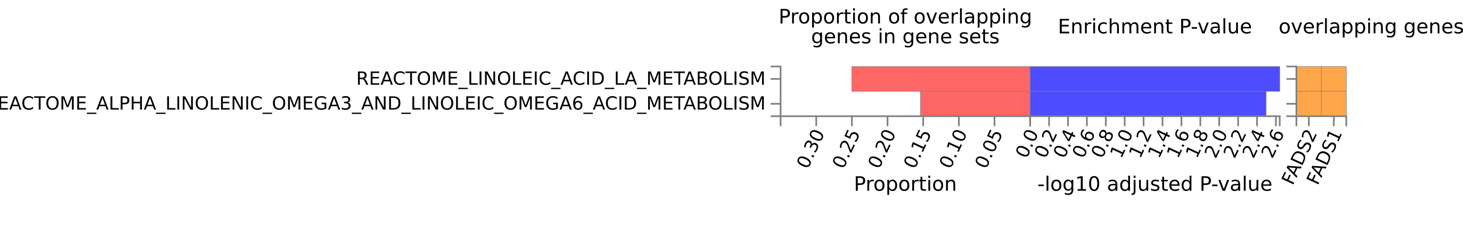


Gene Ontology biological process


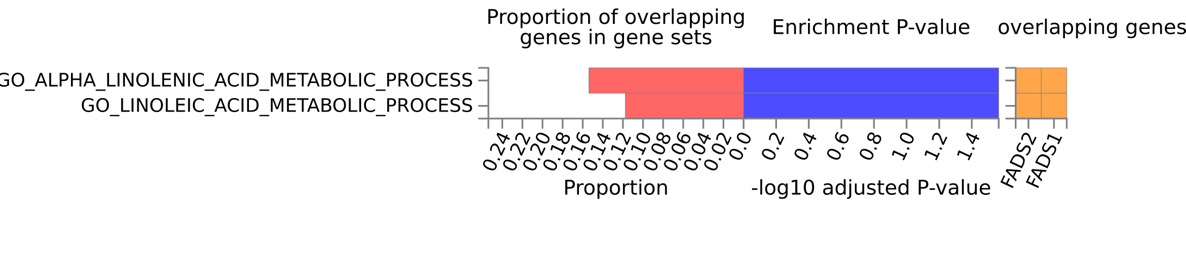


Wikipathways


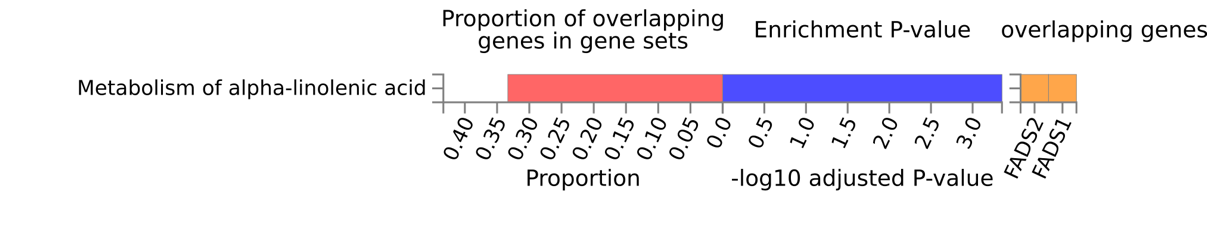


**Figure S3.** Functional analysis of 98 genetic variants included in the polygenic risk score
